# argyle: An R Package for Analysis of Illumina Genotyping Arrays

**DOI:** 10.1534/g3.115.023739

**Published:** 2015-12-18

**Authors:** Andrew P. Morgan

**Affiliations:** Department of Genetics, University of North Carolina, Chapel Hill, North Carolina 27599-7264

**Keywords:** SNP microarrays, genotyping, software

## Abstract

Genotyping microarrays are an important and widely-used tool in genetics. I present argyle, an R package for analysis of genotyping array data tailored to Illumina arrays. The goal of the argyle package is to provide simple, expressive tools for nonexpert users to perform quality checks and exploratory analyses of genotyping data. To these ends, the package consists of a suite of quality-control functions, normalization procedures, and utilities for visually and statistically summarizing such data. Format-conversion tools allow interoperability with popular software packages for analysis of genetic data including PLINK, R/qtl and DOQTL. Detailed vignettes demonstrating common use cases are included as supporting information. argyle bridges the gap between the low-level tasks of quality control and high-level tasks of genetic analysis. It is freely available at https://github.com/andrewparkermorgan/argyle and has been submitted to Bioconductor.

High-throughput genotyping of tens of thousands of single nucleotide polymorphisms (SNPs) using microarrays is common practice in both laboratory and population genetics. Genotypes at a dense panel of biallelic markers with a low rate of missing data are a valuable resource for breeding, marker-assisted selection, genetic mapping, and analyses of population structure. The Illumina Infinium system is one popular and cost-effective (approximately $100/sample) platform. Custom Illumina arrays are available for many organisms of research, agricultural, or ecological interest including mouse (Morgan *et al.* 2015, this issue), dog, cat (Willet and Haase 2014), chicken, cow, pig, horse, sheep (Kijas *et al.* 2009), salmon (Johnston *et al.* 2013), and cotton (Hulse-Kemp *et al.* 2015).

Infinium arrays consist of many thousands of short invariant oligonucleotide probes conjugated to silica beads. Sample DNA is hybridized to the probes and a single-base, hybridization-dependent extension reaction is performed at the target SNP. Alternate alleles (herein denoted A and B) are labeled with different fluorophores (Steemers *et al.* 2006). Raw fluorescence intensity from the two color channels is processed into a discrete genotype call at each SNP, and both the total intensity from both channels, and the relative intensity in one channel *vs.* the other, are informative for copy number.

Many tools, both open-source and proprietary, already exist for postprocessing of raw hybridization intensity data. R packages include beadarray (Dunning *et al.* 2007), lumi (Du *et al.* 2008), and crlmm (Ritchie *et al.* 2009) among others. Illumina’s proprietary BeadStudio software is widely used by commercial laboratories and core facilities. BeadStudio applies a six-step “affine normalization” (Peiffer 2006), which pools data across many probes and many arrays. Intensities from the two color channels (herein denoted *x* and *y*) are transformed to lie in the standard coordinate plane, with homozygous genotypes near the *x* and *y* axes, heterozygous genotypes approximately on the x=y diagonal, and R=x+y≈1. Biallelic genotypes are then called by clustering in this space.

Fewer tools exist for downstream quality control, exploratory analysis and interpretation of genotype calls jointly with underlying hybridization intensity data. To fill this gap, I present argyle, a package for the R statistical computing environment. The purpose of argyle is to provide simple and flexible tools for programmatic access to data from SNP arrays, with an emphasis on visualization. Although some functionality is tailored to Illumina arrays, many of the features are general enough to accommodate any dataset that can be expressed as a matrix of genotypes at biallelic markers. The main text of this paper outlines the key features of argyle; detailed code vignettes are provided as supplementary material.

## Methods

The design of argyle is inspired by the PLINK software [https://www.cog-genomics.org/plink2/; Purcell *et al.* (2007)]. A PLINK fileset has three parts: a genotype matrix, a marker map, and a “pedigree” (sample and family metadata) file. Likewise, the central data structure in argyle (the genotypes object) stores a matrix of genotype calls, and hybridization-intensity data when available, in parallel with a marker map and sample metadata. A genotypes object is therefore a self-contained and largely self-describing representation of a genotyping dataset. Installation of the package is described in Supporting Information, File S1, and the genotypes object is described in further detail in File S2.

This package explicitly favors *simplicity* and *readability* of code over raw efficiency. It is appropriate for the “medium-sized” data—tens of thousands of markers and hundreds of individuals—regularly encountered in experimental contexts. Users with larger datasets such as those routinely collected in human genetics—millions of markers and thousands of individuals—that do not fit comfortably in memory should explore more sophisticated R packages (such as the GenABEL suite: http://www.genabel.org/).

### Data availability

Source code for argyle and example datasets used to generate the figures in this manuscript are available on GitHub: https://github.com/andrewparkermorgan/argyle.

## Quality Control

**Figure 1:**
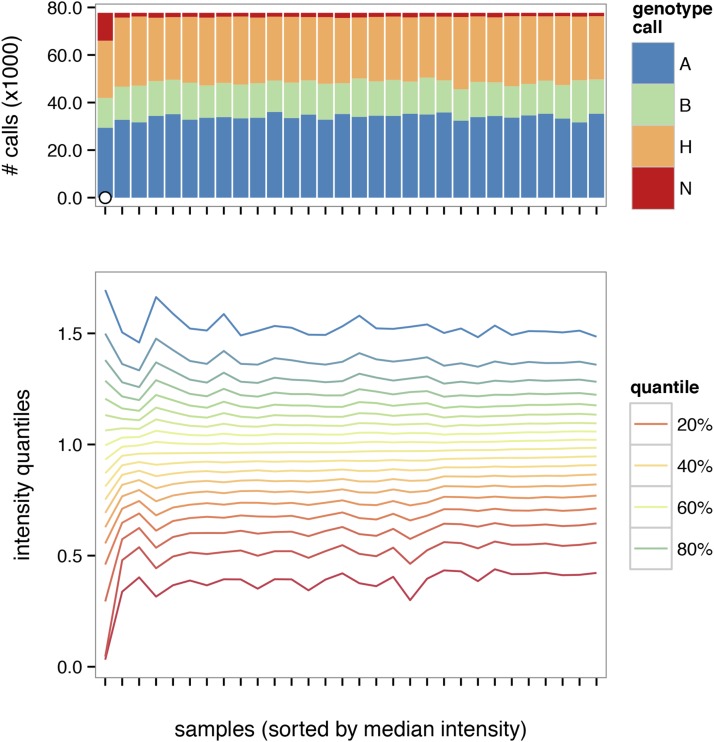
Quality-control summary plot. Distribution of genotype calls is shown in upper panel, and a contour plot of intensity distributions across samples is shown in lower panel. Samples failing quality thresholds are marked with an open dot in the upper panel.

Removal of poorly-performing markers and poor-quality samples is an important precursor to genetic analysis. Failed arrays are characterized by aberrant intensity distributions, excess of missing and heterozygous calls, or both. A summary plot (Figure 1) facilitates the identification of low-quality samples. Concordance between biological sex and sex inferred from calls on the sex chromosomes is also useful for identifying contaminated or swapped samples. Failed arrays can be flagged and removed using global or subgroup-specific thresholds. See File S3 for a worked example.

**Figure 2:**
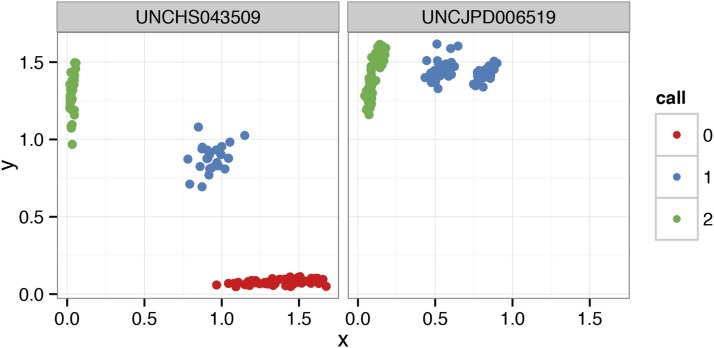
Cluster plots for individual markers. Each point represents a single sample; points are colored according to genotype call, expressed as number of copies of the nonreference allele. The marker on the left performs as expected: the three canonical clusters are present in the expected locations. The marker on the right may be genotyped incorrectly: the homozygous reference cluster (red) is missing, and the nominally heterozygous samples (blue) fall into two clusters. This marker merits further inspection. For example, one nominally heterozygous cluster may correspond to homozygosity for the reference allele or, the marker may be detecting paralogous variation at off-target loci.

**Figure 3:**
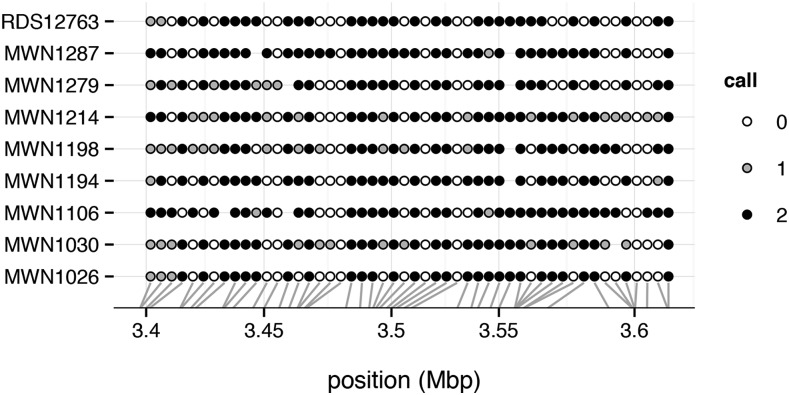
Dotplot representation of genotypes among nine wild-caught mice on proximal chromosome 19 (from Yang *et al.* 2011). Genotype calls are coded as counts of the reference allele, and points are colored according to genotype call. Blank spaces indicate missing calls. Markers are plotted with constant spacing in the main panel; gray lines indicate physical position along the chromosome in megabases (Mbp).

In addition to global summaries, argyle provides easy access to hybridization intensity data from individual probes. Inspection of “cluster plots” for individual probes is useful for confirming the accuracy of genotype calls and diagnosing poorly-performing markers (Figure 2). A dotplot (Figure 3) permits direct inspection of genotype calls at multiple markers over small genomic regions.

## Array Normalization

Illumina BeadStudio uses an “affine normalization” algorithm to perform within- and between-array adjustments to *x*- and *y*-hybridization intensities before calling genotypes. However, further normalization is helpful for analyses of sample contamination and copy number. Two standard metrics are the $\log_{2}R/R_{0}$ ratio (LRR), which captures total hybridization intensity (*R*) relative to a reference level ($R_{0}$); and B-allele frequency (BAF), which captures the relative signal from the A and B alleles (Peiffer 2006). For an uncontaminated euploid sample, the expected value of LRR is 0, and the expected value of BAF is 0.5 at heterozygous markers.

**Figure 4:**
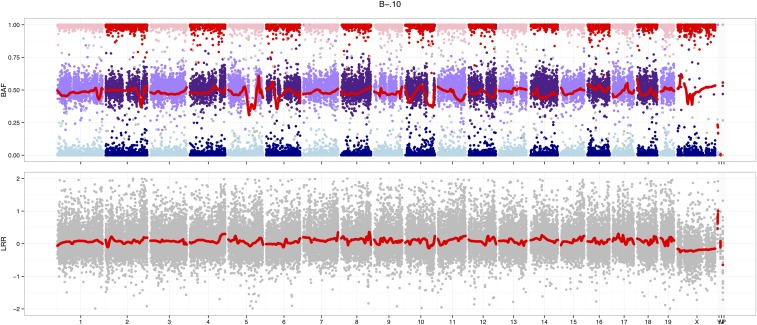
Joint plot of B-allele frequency (BAF, upper panel) and log2 intensity ratio (LRR, lower panel) for an outbred male mouse. The autosomes are almost entirely heterozygous, while the X chromosome is hemizygous: no points appear near BAF = 0.5 on the X chromosome and its LRR is decreased relative to the autosomes. Red traces are a local smoothing of underlying points.

The argyle package implements the thresholded quantile normalization (tQN) approach described in Staaf *et al.* (2008) and Didion *et al.* (2014). Briefly, tQN performs within-array quantile normalization of the *x* channel against the *y* channel to account for dye biases specific to the Infinium chemistry, but places an upper bound on the difference between normalized and unnormalized intensity values. LRR and BAF are then computed using known cluster positions computed from a set of reference samples. The tQN procedure may optionally be preceded by preliminary between-array quantile normalization using routines implemented in the preprocessCore package (Bolstad *et al.* 2003). A joint plot of BAF and LRR (Figure 4) is valuable for assessing heterozygosity, ploidy, sample purity, and sex-chromosome karyotype.

Copy-number inference from Illumina arrays is a well-studied problem for which good solutions already exist—for instance, the standalone software PennCNV (Wang *et al.* 2007), or the R package genoCN (Sun *et al.* 2009). Most of these packages take BAF and LRR values as input and so are easily integrated downstream of argyle.

Systematic batch effects on intensity distributions are possible when analyzing samples processed that were not processed concurrently. The reliability of discrete genotype calls may be unchanged between batches, but downstream analyses that make use of hybridization intensities [*e.g.*, copy-number analyses, or hidden Markov models (HMM) for haplotype inference in multiparental populations (Fu *et al.* 2012; Gatti *et al.* 2014)] may benefit from a further batch correction. One possibility, given *k* nonoverlapping batches, is quantile normalization of batches 1,…,k−1 against the *k*th batch. Although between-batch normalization is not yet implemented in argyle, it is slated for inclusion in future releases.

## Genetics Tools

Utilities are provided for efficient calculation of allele frequencies, heterozygosity and missingness by sample and by marker. When genotypes of both parents and offspring are available, pedigree relationships can be confirmed via checks for Mendelian inconsistencies. Separate datasets can be concatenated or merged using functions that ensure consistency of allele encoding and detect strand swaps [*e.g.*, an (A/G) *vs.* a (T/C) SNP].

**Figure 5:**
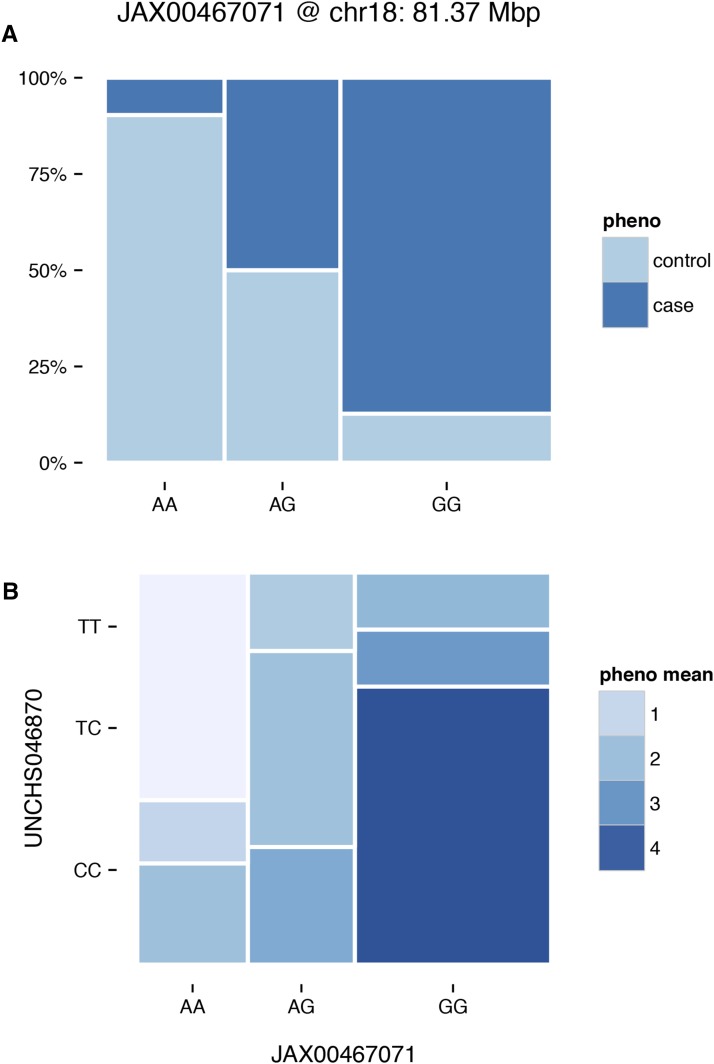
(A) One-way mosaic plot. Width of each bar is proportional to the frequency of the corresponding genotype; fill colors indicate phenotype, here case or control status. (B) Two-way mosaic plot. Area of each block is proportional to two-locus genotype frequency, and fill colors indicate phenotype mean for each two-locus genotype.

To facilitate analysis of genotypes from experimental crosses, argyle provides functions for recoding alleles with respect to parental lines. A general-purpose HMM allows for reconstruction of haplotype mosaics, given a panel of reference samples and a genetic map—although users are cautioned that more sophisticated implementations are available for some special cases (Broman *et al.* 2003; Fu *et al.* 2012; Gatti *et al.* 2014). Mature tools for genetic mapping in the R environment already exist (*e.g.*, R/qtl; Broman *et al.* 2003). Genotypes processed with argyle can be readily converted to R/qtl format to create a unified pipeline for quantitative-trait locus (QTL) mapping. One- and two-locus “mosaic plots” allow joint visualization of allele frequencies and phenotype at candidate QTL (Figure 5). A worked example is provided in File S4.

**Figure 6:**
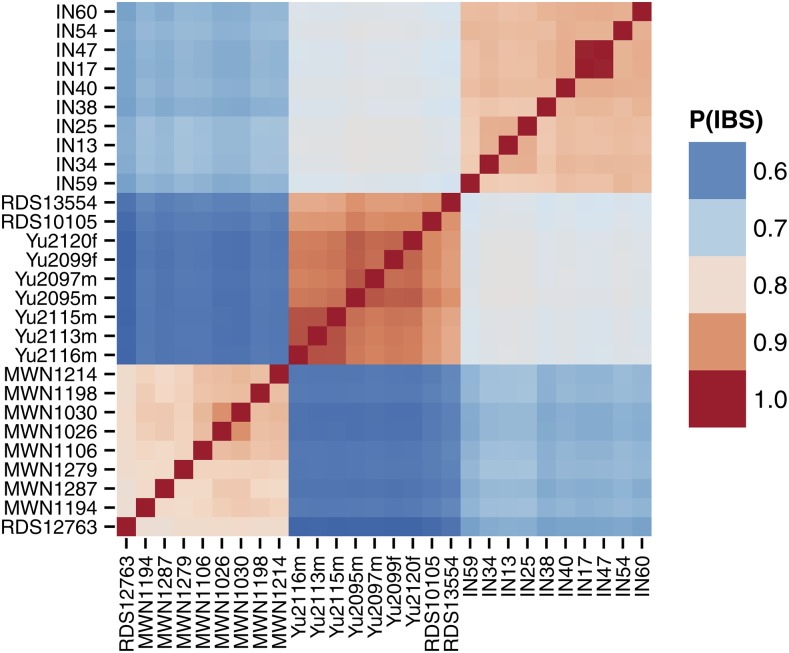
Heatmap representation of matrix of pairwise genetic distances between 28 wild-caught mice from three different subspecies using data from the Mouse Diversity Array (Yang *et al.* 2011). Genetic distance is defined here as the proportion of alleles shared identical by state between two individuals. The matrix is hierarchically clustered to that more closely-related samples are adjacent to each other. The heatmap is useful for visualizing population structure; here it reveals obvious genetic differentiation between mouse subspecies.

**Figure 7:**
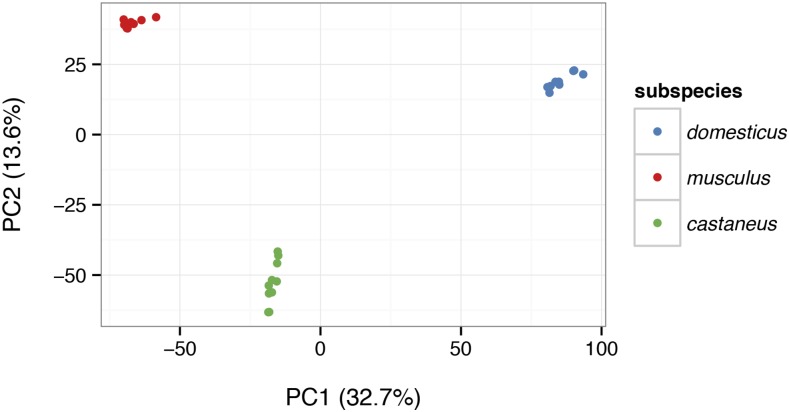
Projection of the same 28 samples from Figure 6 onto the top two principal components (PCs) of the genotypes matrix. The block structure of the kinship matrix corresponds to the three clusters revealed by principal component analysis (PCA), which in turn correspond to three distinct subspecies.

Genome-wide patterns of relatedness can be explored using built-in functions for efficient kinship estimation (Figure 6) and principal components analysis (Figure 7). See File S5 for more detailed demonstration of functions useful for population-genetic analysis.

## Data Export

The argyle package provides functions to convert genotypes objects to other formats either within the R session (for R/qtl and DOQTL) or on disk. Currently argyle supports export to either PLINK binary format (*.fam/*.bim/*.bed) or Stanford HGDP format. PLINK provides command-line utilities to convert its file format to many others, including VCF, LINKAGE (*.map/*.ped), Haploview, STRUCTURE, and fastPHASE. In addition, since genotypes objects are regular R matrices, users can adapt them to bespoke input formats required by other tools for genetic analysis.

## Performance

**Figure 8:**
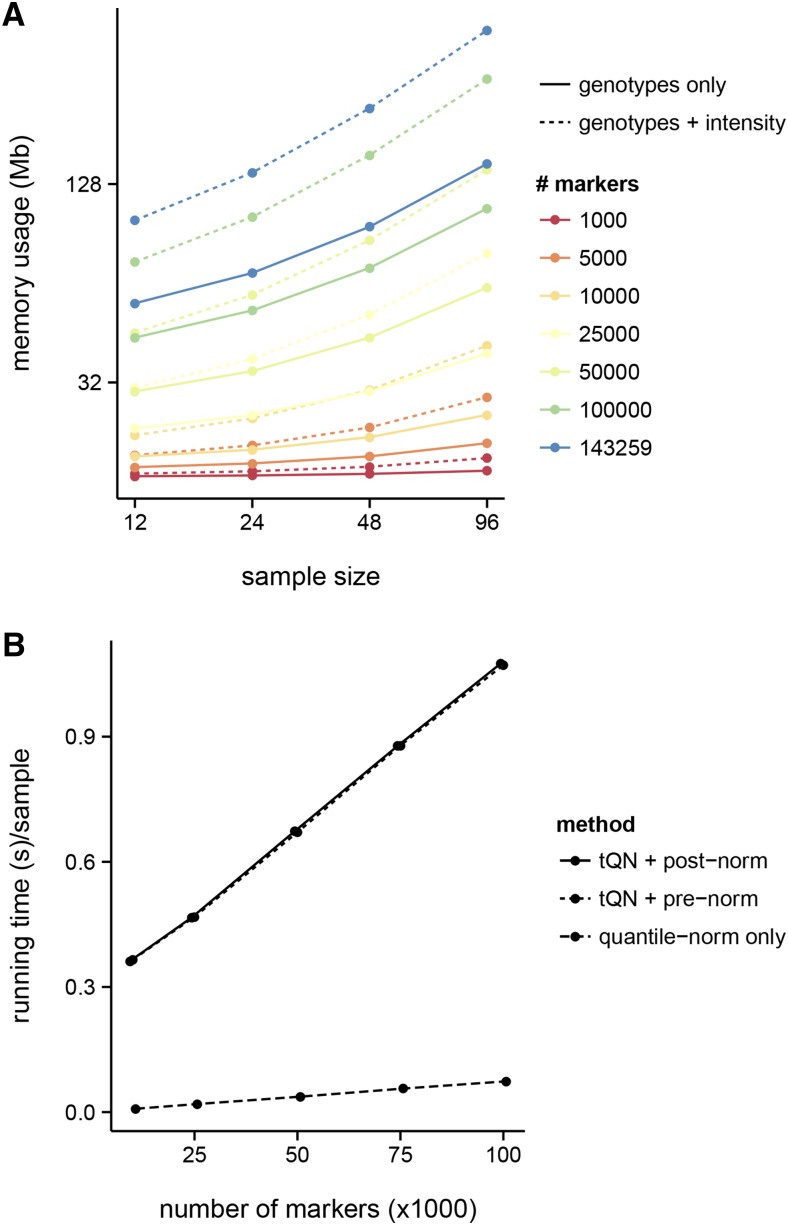
(A) Memory requirements for genotypes objects (estimated via R’s object.size() with varying numbers of markers and samples, with and without hybridization intensities. (B) Running time per sample of the thresholded quantile normalization (tQN) procedure, with either initial quantile normalization or postpolishing, compared to quantile normalization alone.

argyle and its dependencies are compatible with R (≥2.14) on Windows or Mac OS X. The performance of argyle benefits from optimized code in several existing R packages including data.table and Rcpp (Eddelbuettel 2013). Reading a dataset of realistic size – 96 samples × 77,808 markers (164 Mb ZIP-compressed on disk)—from Illumina BeadStudio output into an R session takes about 30 sec. The full dataset, including hybridization intensities and sample and marker metadata, occupies 202.4 Mb; without hybridization intensities, the size drops to 77.9 Mb. Memory usage scales approximately linearly with either the number of samples or the number of markers (Figure 8A). The most computationally-intensive component of argyle is the tQN procedure, and is implemented in C++. Its running time is compared to the quantile normalization routine from the preprocessCore package in Figure 8B. These resource requirements are well within the range of a typical laptop or desktop computer.

R’s internal limit of $2^{31}-1$ entries for any matrix or vector places an upper bound on the dimensions of a genotypes object. For arrays with between 10,000 and 150,000 markers, this translates to a limit of between 14,000 and 21,000 samples.

Tests were performed in R 3.1.2 (64-bit) on a MacBook Air, with a single 1.7 Ghz Intel Core i7 processor, and 8 Gb RAM.

## Supplementary Material

Supporting Information
