# Supplementary material for: argyle: An R Package for Analysis of Illumina Genotyping Arrays
Source: G3 (Bethesda). 2015 Dec 18;6(2):281–6. doi: 10.1534/g3.115.023739 (PMC4751548; doi:10.1534/g3.115.023739)
Supplement: Supporting Information [file supp_6_2_281__index.html]

argyle: An R Package for Analysis of Illumina Genotyping Arrays — Supporting Information 

# argyle: An R Package for Analysis of Illumina Genotyping Arrays

## Supporting Information for Morgan, 2016

**Files in this Data Supplement:**

- File S1 - Installation. (.pdf, 177 KB)
- File S2 - Data import. (.pdf, 223 KB)
- File S3 - Quality control. (.pdf, 318 KB)
- File S4 - Analysis of an experimental cross. (.pdf, 425 KB)
- File S5 - Analysis of genotypes from natural populations. (.pdf, 285 KB)
