# Supplementary material for: argyle: An R Package for Analysis of Illumina Genotyping Arrays
Source: G3 (Bethesda). 2015 Dec 18;6(2):281–6. doi: 10.1534/g3.115.023739 (PMC4751548; doi:10.1534/g3.115.023739)
Supplement: Supporting Information [file supp_g3.115.023739_FileS1.pdf]

Installing `argyle`

Andrew P Morgan

2015-10-08

Dependencies

The following packages are *required* for the core routines (import, quality checks, internal utilities) in `argyle`. From CRAN:

- `Rcpp`
- `data.table` (>= 1.9.4)
- `digest`
- `reshape2`
- `plyr`

And from Bioconductor:

- `preprocessCore`

The next set of dependencies is *suggested* – the package will install without them, but not all its functions will work. In particular the plotting functions rely on `ggplot2` and friends (all available from CRAN):

- `ggplot2`
- `grid`
- `gridExtra`
- `gtable`
- `RColorBrewer`

And, finally, the principal components analysis function benefits from the speedups to matrix factorization implemented in `corpcor`.

Option 1: pre-compiled binary

First download the pre-compiled binary appropriate for your operating system from the `argyle` GitHub repository: https://github.com/andrewparkermorgan/argyle. Assume that it's called `argyle_0.1.tgz`.

Until `argyle` is available on CRAN, dependencies must be installed by hand. To do this, first tell `R` to look for packages in the United States CRAN mirror (and avoid the "choose a mirror" pop-up window).

```
repos <- c("http://cran.us.r-project.org")
```

Bioconductor provides a custom installer function, which can be installed with

```
source("http://bioconductor.org/biocLite.R")
```

Then use `install.packages()` to get CRAN dependencies (and *their* dependencies, recursively).

```
depends <- c("Rcpp","data.table","digest","reshape2","plyr")
suggests <- c("ggplot2","grid","gridExtra","gtable","RColorBrewer","corpcor")
install.packages( c(depends, suggests), repos = repos )
```

And the single Bioconductor dependency.

```
biocLite("preprocessCore")
```

Finally, install `argyle` from the downloaded bundle.

```
install.packages("~/Dropbox/pmdvlab/argyle_0.1.tgz", repos = NULL)
```

Option 2: from GitHub source

The latest version of `argyle` can be installed quickly and easily from source code on GitHub using the `devtools` package. (Installing `devtools` and its dependencies – particularly the `Xcode` command-line tools on Mac OSX, or equivalently, `Rtools` for Windows – may be time-consuming for novice users.)

In any case, installation from GitHub requires a single command:

```
devtools::install_github("andrewparkermorgan/argyle")
```

Building the package from source will involve compiling some `C++` code. No promises are made for users with idiosyncratic library paths, etc.
