# Supplementary material for: argyle: An R Package for Analysis of Illumina Genotyping Arrays
Source: G3 (Bethesda). 2015 Dec 18;6(2):281–6. doi: 10.1534/g3.115.023739 (PMC4751548; doi:10.1534/g3.115.023739)
Supplement: Supporting Information [file supp_g3.115.023739_FileS2.pdf]

Importing raw genotype data with `argyle`

Andrew P Morgan

2015-10-07

Introduction

This vignette demonstrates the steps required to load genotype data from output files produced by Illumina BeadStudio. Two files are expected: one named like `*Sample_Map.zip`, the sample manifest, and one named like `*FinalReport.zip`, which contains genotype calls and hybridization intensity values. These are plain-text reports compressed with ZIP. For Mac and Linux users, they will be decompressed on-the-fly by `argyle`; Windows users will need to unzip them before starting the import process.

The `FinalReport` is expected to have the following columns: `SNP Name` (unique marker name), `Sample ID` (unique sample name), `Allele1 - Forward` (first allele call, one of `ACGTN`), `Allele 2 - Forward` (second allele call, one of `ACGTN`), `X` (hybridization intensity in the x dimension) and `Y` (intensity in the y dimension). Column names are preceeded by a header section describing the number of samples and markers genotyped. Each row after the column names represents a single genotype (*ie.* a sample-marker pair). Homozygous calls will have the same value in columns `Allele 1` and `Allele 2`; heterozygous calls will have diffferent values in the two columns; and missing calls will have an `N` in both columns. Allele calls are made with respect to the forward strand for ease of comparison with public databases and reference genome sequences.

In addition to the `Sample_Map` and `FinalReport`, `argyle` requires the user to supply a marker map for the array. This should be a `dataframe` with at least the following six columns: `chr` (chromosome), `marker` (unique marker name, to be matched to `SNP Name` in `FinalReport`), `cM` (genetic position in centimorgans), `pos` (physical position in base pairs), `A1` (reference allele) and `A2` (alternate allele). Alleles should be specified with respect to the forward strand, as in `FinalReport`. The `dataframe` should have row names, and these should match the values in the `marker` column.

> The marker map must be prepared ahead of time, and doing so requires knowledge of the array platform. Maps for the Mouse Universal Genotyping Array series are available for download at http://csbio.unc.edu/CCstatus/.

This and other vignettes included with `argyle` makes use of genotyping reports from the Mouse Universal Genotyping Arrays for the house mouse (*Mus musculus*) sold by Neogen Inc. Users of other platforms should consult their vendor with specific questions about file formats.

Example dataset

We use an example dataset from the MegaMUGA array consisting of 96 samples x 77808 markers. It consists of two files, residing in `~/argyle/datasets/mega_example`:

```
ls -lh ~/argyle/datasets/mega_example
```

```
## total 321224
## -rw-r--r--@ 1 apm  staff   157M Oct  7 15:24 FinalReport.zip
## -rw-r--r--@ 1 apm  staff   5.0K Oct  7 15:55 Sample_Map.txt
```

Importing from BeadStudio files

First, load the `argyle` package and (for convenience) set the working directory for the `R` session.

```
library(argyle)
setwd("~/argyle")
```

Next load the marker map, which has been pre-constructed and saved in an `Rdata` file. The object is a `dataframe` called `snps`.

```
load("snps.megamuga.Rdata")
head(snps)
```

```
##               chr      marker     cM     pos A1 A2
## UNC6         chr1        UNC6 1.4995 3000355  T  C
## JAX00000010 chr1 JAX00000010 1.5620 3125499  A  G
## JAX00240603 chr1 JAX00240603 1.6075 3242877  C  T
## JAX00240610 chr1 JAX00240610 1.6111 3256689  C  T
## JAX00240613 chr1 JAX00240613 1.6260 3313481  T  C
## JAX00240636 chr1 JAX00240636 1.6433 3379644  C  A
```

To import genotypes, use the `read.beadstudio()` function. Argument `in.path` specifies the directory in which `argyle` will search for the `Sample_Map` and `FinalReport` files, and `prefix` gives the file name prefix (in our case, none). The marker map (argument `snps`) must also be provided. Genotypes at markers not present in the marker map will be omitted from the output. If `keep.intensity = TRUE` (the default), hybridization intensities will be imported along with genotype calls.

```
geno <- read.beadstudio(prefix = "", in.path = "./datasets/mega_example", snps = snps, keep.intensity =
```

```
## Reading sample manifest from < ./datasets/mega_example/Sample_Map.txt > ...
## Reading genotypes and intensities for 77808 markers x 96 samples from < ./datasets/mega_example/Fina
## Constructing genotype matrix...
## Constructing intensity matrices...
##   77808 sites x 96 samples
## Done.
```

The function returns a `genotypes` object with the stated dimensions. Depending on hardware and operating system, a dataset of this size (~ 150 Mb ZIP-compressed) can be expected to take less than one minute.

The genotypes object

The `genotypes` object is the central data structure of the `argyle` package. The design mimics the file structure used by `PLINK` (see here for details). A `genotypes` is a matrix with markers in rows and samples in columns; each entry is a biallelic genotype call. Row names match marker names, and column names match sample names. The `genotypes` class inherits from the `matrix` in base `R`, so all functions which work for matrices – `apply()`, `dim()` and so on – will also work for `genotypes` objects. Marker and sample metadata are stored as attributes of the matrix.

For a high-level overview `genotypes` object, use `summary()`:

```r
summary(geno)
```

```
## --- geno ---
## A genotypes object with 77808 sites x 96 samples
## Allele encoding: native
## Intensity data: yes (raw)
## Sample metadata: yes ( 0 male / 0 female / 96 unknown )
## Filters set: 0 sites / 0 samples
## File source: /Users/apm/Dropbox/pmdvlab/argyle/manuscript/vignettes/datasets/mega_example (on 2015-1
## Checksum: ccf22694fee67b07e68b8aeb6fb5b053
```

As expected, the object contains 77808 markers and 96 samples. Alleles are encoded using the `native` scheme for BeadStudio – that is, as nucleotides (`ACGT`) with respect to the forward strand for homozygous calls, `H` for heterozygous calls, and `N` for no-calls (missing data). Intensity data is present, and `raw` indicates that no intensity normalization has been applied yet. No filters have been set. A checksum is computed during the import process that can be used to check file integrity if re-importing the same data again.

To peek at the contents of the object, use `head()`:

```r
head(geno)
```

```
## Genotypes matrix:
##                    B-1  B-10 B-101 B-102 B-103 B-104 B-105 B-106 B-107 B-108
##             UNC6     N     H     C     H     H     T     H     C     T     C
##      JAX00000010     H     H     G     H     H     A     H     G     A     G
##      JAX00240603     N     H     T     H     H     C     H     T     H     T
##      JAX00240610     C     C     C     C     C     C     C     C     C     C
##      JAX00240613     H     H     C     H     H     T     H     C     T     C
##      JAX00240636     C     C     C     C     C     C     C     C     H     C
##      JAX00240649     C     C     T     H     C     C     C     C     C     C
##      JAX00000040     G     G     G     G     G     G     G     G     G     G
##     UNC010515443     G     G     H     H     G     G     G     G     H     G
##          UNC9371     C     C     H     C     C     C     C     C     H     C
##
## Marker map:
##    chr       marker     cM     pos A1 A2
##   chr1         UNC6 1.4995 3000355  T  C
##   chr1  JAX00000010 1.5620 3125499  A  G
##   chr1  JAX00240603 1.6075 3242877  C  T
##   chr1  JAX00240610 1.6111 3256689  C  T
##   chr1  JAX00240613 1.6260 3313481  T  C
##   chr1  JAX00240636 1.6433 3379644  C  A
##   chr1  JAX00240649 1.6480 3445065  C  T
##   chr1  JAX00000040 1.6480 3534900  G  A
##   chr1 UNC010515443 1.6480 3658709  G  A
##   chr1      UNC9371 1.6480 3882799  C  T
##
## Sample info:
##     fid    iid mom dad sex pheno
##    B-.1   B-.1   0   0   0    -9
##   B-.10  B-.10   0   0   0    -9
##  B-.101 B-.101   0   0   0    -9
##  B-.102 B-.102   0   0   0    -9
```

```
##  B-.103 B-.103   0   0   0    -9
##  B-.104 B-.104   0   0   0    -9
##  B-.105 B-.105   0   0   0    -9
##  B-.106 B-.106   0   0   0    -9
##  B-.107 B-.107   0   0   0    -9
##  B-.108 B-.108   0   0   0    -9
```

This shows the top corner of the genotypes matrix, and corresponding rows from the marker map and sample metadata. Sample names are truncated to keep the output compact.

To access sample metadata, use `samples()`:

```
head( samples(geno) )
```

```
##           fid    iid mom dad sex pheno
## B-.1     B-.1   B-.1   0   0   0    -9
## B-.10   B-.10  B-.10   0   0   0    -9
## B-.101 B-.101 B-.101   0   0   0    -9
## B-.102 B-.102 B-.102   0   0   0    -9
## B-.103 B-.103 B-.103   0   0   0    -9
## B-.104 B-.104 B-.104   0   0   0    -9
```

This function returns a dataframe with (at least) six columns: `fid` (a "family" identifier, or more generally any grouping variable; it defaults to the sample name), `iid` (unique sample name), `mom` and `dad` (pedigree information, if any; 0 indicates missing data), `sex` (0 = unknown, 1 = male, 2 = female), and `pheno` (phenotype; -9 indicates missing data). Row names match the `iid` column.

Similarly, `markers()` returns the marker map:

```
head( markers(geno) )
```

```
##              chr      marker     cM     pos A1 A2
## UNC6        chr1        UNC6 1.4995 3000355  T  C
## JAX00000010 chr1 JAX00000010 1.5620 3125499  A  G
## JAX00240603 chr1 JAX00240603 1.6075 3242877  C  T
## JAX00240610 chr1 JAX00240610 1.6111 3256689  C  T
## JAX00240613 chr1 JAX00240613 1.6260 3313481  T  C
## JAX00240636 chr1 JAX00240636 1.6433 3379644  C  A
```

The usual `R` slicing and indexing conventions work for `genotypes` objects, and `argyle` ensures that genotype calls, marker metadata, sample metadata, and intensity matrices are all kept in sync. For instance, to extract just the first 10 markers (rows) we can do

```
g1 <- geno[ 1:10, ]
summary(g1)
```

```
## --- g1 ---
## A genotypes object with 10 sites x 96 samples
## Allele encoding: native
## Intensity data: yes (raw)
## Sample metadata: yes ( 0 male / 0 female / 96 unknown )
## Filters set: 0 sites / 0 samples
```

See that g1 is now a genotypes object of size 10×96. Furthermore, we can use `subset()` to easily perform more complex filtering. For instance, to extract all markers in the first 50 Mbp of chromosome 1:

```
g2 <- subset(geno, chr == "chr1" & pos < 50e6)
nrow(g2) # how many markers are left?
```

```
## [1] 1247
```

Note that `argyle`'s `subset()`, like `subset.data.frame()` in base `R`, uses *lazy evaluation*. By default the subsetting expression is evaluated in the context of the marker map, but we can also subset according to sample properties by passing the argument `by = "samples"`:

```
g3 <- subset(geno, fid != "exclude_me", by = "samples")
ncol(g3) # how many samples are left?
```

```
## [1] 96
```

Allele encoding schemes

When reading genotypes from BeadStudio output, alleles are encoded just as they appear in the file (`native` mode). However, it is often useful to represent genotypes as allele counts (of the non-reference or minor allele). To convert to numeric allele encoding with 0 = homozygous reference allele, 1 = heterozygous and 2 = homozygous alternate allele, do

```
g2.recode <- recode(g2, "01")
```

```
## Recoding to 0/1/2 using reference alleles.
```

```
summary(g2.recode)
```

```
## --- g2.recode ---
## A genotypes object with 1247 sites x 96 samples
## Allele encoding: 01
## Intensity data: yes (raw)
## Sample metadata: yes ( 0 male / 0 female / 96 unknown )
## Filters set: 0 sites / 0 samples
```

See that the allele encoding is now listed as the `01` mode. In this mode no-calls (`N`) are converted to true missing values (`NA`).

In the numeric encoding it is easy and fast to compute allele frequencies.

```
af <- rowMeans(g2.recode, na.rm = TRUE)/2
quantile(af, na.rm = TRUE)
```

```
##        0%       25%       50%       75%      100%
## 0.0000000 0.1875000 0.3854167 0.5729167 1.0000000
```

If needed we can revert to the `native` nucleotide encodings without loss of information.

```r
g2.recode.again <- recode(g2.recode, "native")
```

```
## Recoding to character using reference alleles.
```

```r
identical(g2, g2.recode.again)
```

```
## [1] TRUE
```

Other input formats

> The `argyle` package can read `PLINK` binary filesets, and by extension, any format that can be converted by `PLINK` into such a fileset. `PLINK`-related functions are covered in a separate vignette.

Users whose genotype data is stored in ad hoc formats can still benefit from the functions provided by `argyle`. If genotype data can be manipulated into matrix form in `R`, a `genotypes` object can be constructed manually as shown in the example below.

```r
# grab a few SNPs from existing map
my.snps <- snps[ 1:10, ]

# fake some genotypes (all homozygous for ref allele)
x <- matrix(0, nrow = 10, ncol = 5)

# genotype matrix *must* have row and column names
my.samples <- paste0("sample", 1:5)
rownames(x) <- rownames(my.snps)
colnames(x) <- my.samples

# now roll it up into a `genotypes` object
g <- genotypes(x, snps, alleles = "01")
summary(g)
```

```
## --- g ---
## A genotypes object with 10 sites x 5 samples
## Allele encoding: 01
## Intensity data: no
## Sample metadata: yes ( 0 male / 0 female / 5 unknown )
## Filters set: 0 sites / 0 samples
```
