# Supplementary material for: argyle: An R Package for Analysis of Illumina Genotyping Arrays
Source: G3 (Bethesda). 2015 Dec 18;6(2):281–6. doi: 10.1534/g3.115.023739 (PMC4751548; doi:10.1534/g3.115.023739)
Supplement: Supporting Information [file supp_g3.115.023739_FileS3.pdf]

Quality control of array genotyping data with `argyle`

Andrew P Morgan

2015-10-08

Introduction

Proper quality control of array genotypes is an important prerequisite to further analysis. Genotype quality can be assessed on two dimensions: markers (sites) or samples. Both are important, but in general sample-level quality checks must be performed first, in order to obtain a cleaned dataset useful for assessing the performance of specific markers.

Sample-level quality checks are implemented at three levels in `argyle`:

- distribution of hybridization intensities
- distribution of genotype calls
- biological constrants (concordance between biological sex *vs* sex-chromosome genotypes; heterozygosity; ...)

Failed arrays have onr or more of the following properties, each of which will be discussed in further detail in subsequent sections:

- aberrant distribution of hybridization intensities (higher variance but asymmetric, skewed towards low intensities)
- excess of no-calls and heterozygous relative to homozygous calls genome-wide
- sex-chromosome calls discordant with biological sex

For datasets of sufficient size (say, at least 24 samples), these quality checks can be performed without an external reference panel of known good samples: unless an entire batch has failed, failed samples should be outliers with respect to good samples within the batch. In this vignette we use a dataset from 96 Diversity Outbred (**???**) mice genotyped on the MegaMUGA array.

Primer on hybridization intensities for Illumina arrays

What follows is a very brief review of the character of hybridization-intensity data produced by Illumina arrays. For a full discussion see (Steemers *et al.* 2006).

Illumina arrays, unlike Affymetrix platforms, are printed with invariant oligonucleotide probes. The two possible alleles at the target SNP are distinguished via a single-base extension reaction with fluorescent-conjugated nucleotides. At a given SNP marker, fluorescence signals are recorded in two channels (denote them x and y) for every sample. The values recorded on the instrument are *raw* intensities, which we will denote x_0 and y_0. The Illumina BeadStudio software uses an "affine polish" algorithm pools information from many samples and many arrays to transform $x_0 \rightarrow x$ and $y_0 \rightarrow y$, where the *transformed* intensities $x, y \geq 0$ and the total intensity $R = x + y \approx 1$. Samples homozygous for allele A are expected to form a cluster near $(1, 0)$; those homozygous for allele B near $(1, 0)$; and heterozygous samples near the $1:1$ diagonal at a distance of ≈ 1 from the origin. Samples falling near the origin – *ie.* those with low total hybridization intensity – will be no-calls. A clustering algorithm is applied to the transformed intensities to yield discrete genotype calls.

A consequence of the affine intensity transformation is that, at any given marker, samples with non-missing calls are expected to have total intensity ≈ 1 regardless of their genotype call. Although R is often used as

the measure of total intensity, `argyle` defines the alternative parameter $d = \sqrt{x^2 + y^2}$ (the distance from the origin in intensity space). The distribution of d across all markers for each sample is a sensitive indicator of its overall quality. We use d rather than R because it is less prone to overestimation of total hybridization signal in highly heterozygous samples (cf. the triangle inequality).

Data import

First load `argyle` and set the working directory.

```
library(argyle)
setwd("~/argyle")
```

Load the marker map for MegaMUGA.

```
load("snps.megamuga.Rdata")
```

Import genotypes from BeadStudio output files, and check that import was successful.

```
geno <- read.beadstudio(prefix = "", in.path = "./datasets/mega_example", snps = snps, keep.intensity =
```

```
## Reading sample manifest from < ./datasets/mega_example/Sample_Map.txt > ...
## Reading genotypes and intensities for 77808 markers x 96 samples from < ./datasets/mega_example/Fina
## Constructing genotype matrix...
## Constructing intensity matrices...
##   77808 sites x 96 samples
## Done.
```

Subsequent quality checks run much faster if genotypes have been converted to numeric encoding (*ie.* allele counts), which we will do before proceeding.

```
geno <- recode(geno, "01")
```

```
## Recoding to 0/1/2 using reference alleles.
```

Quality annotation in `argyle`

Marker- and sample-level filters are recorded as attributes of a `genotypes` object. Every marker and every sample has a filter string which is initialized empty. Filters are indicated by one-letter codes – `H` for excess heterozygosity; `N` for excess no-call rate; `I` for aberrant intensity patterns; and `F` for other – which are added to the filter string as appropriate. Sites or samples failing quality checks are thus flagged but are *not* removed until explicity requested by the user.

Sample-level QC

Sample-level quality checks are implemented in a single wrapper function, `run.sample.qc()`. This function computes the distributions of intensities and genotype calls per sample. If any of the filtering criteria listed below are specified, samples failing the filters will be flagged but *not* removed from the dataset.

- `max.N` – upper threshold for count of no-calls per sample
- `max.H` – upper threshold for count of heterozygous calls per sample

These parameters can be specified as either scalars or named lists. In the former case, the same threshold is applied to all samples. In the latter case, thresholds are applied in groups defined by the `fid` column in the sample metadata. For Diversity Outbred mice genotyped on MegaMUGA, 4000 no-calls and 40000 heterozygous calls are reasonable upper limits.

The function returns a copy of the input `genotypes` object with the results of quality checks attached as an attribute.

```
geno <- run.sample.qc(geno, max.N = 4e3, max.H = 40e3)
```

```
## Performing QC checks on genotype calls...
## Performing QC checks on hybridization intensities...
## 0 markers and 1 samples now flagged as low-quality.
```

```
summary(geno)
```

```
## --- geno ---
## A genotypes object with 77808 sites x 96 samples
## Allele encoding: 01
## Intensity data: yes (raw)
## Sample metadata: yes ( 0 male / 0 female / 96 unknown )
## Filters set: 0 sites / 1 samples
```

Note that 1 samples are now flagged as low-quality. To see more detail about which samples failed which filters, we can do

```
summarize.filters(geno)
```

```
##   sites samples
## N     0       1
## H     0       0
## I     0       0
## F     0       0
```

We see that 1 samples are flagged for having too many missing calls, and 0 for having too many heterozygous calls.

A graphical summary of quality checks can be obtained using `qcplot()`. When quality checks have been pre-computed, they will be plotted immediately; if not, sample-level checks will be run first and the result plotted.

```
qcplot(geno)
```

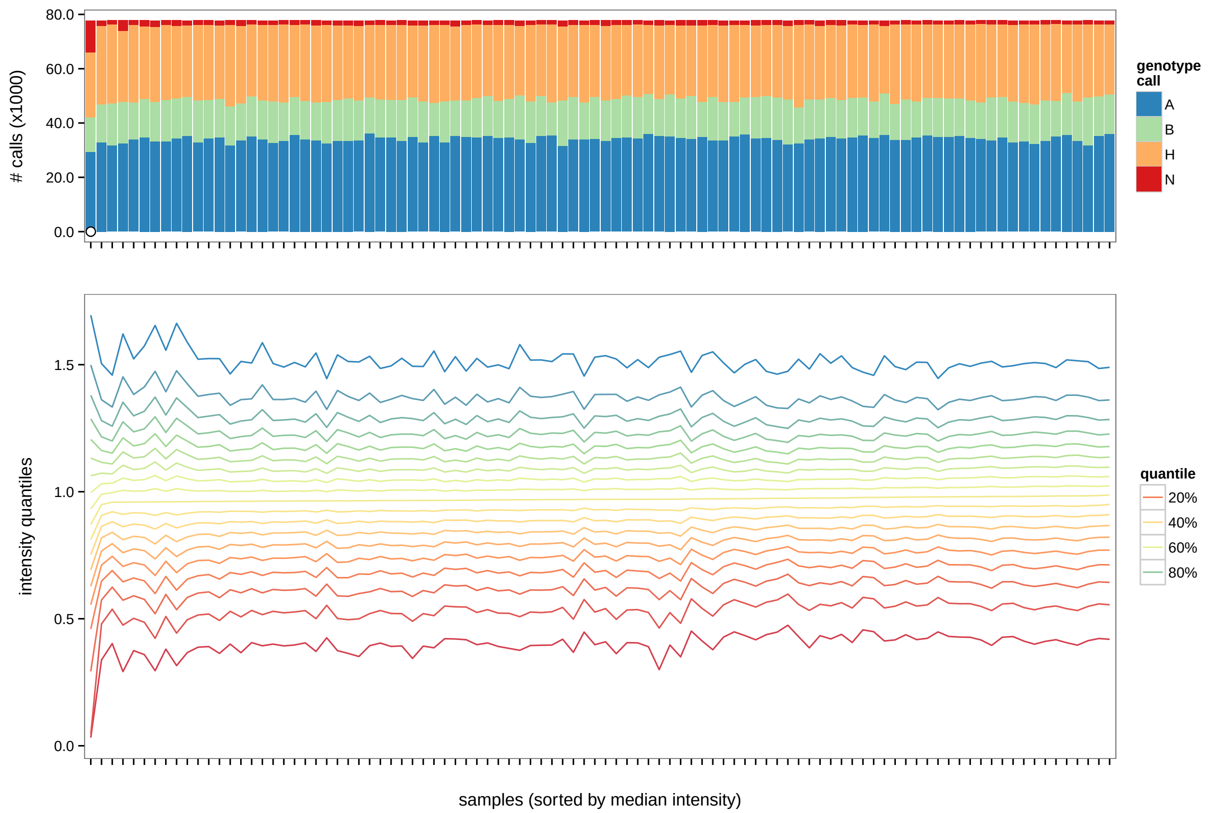

The top panel of this plot displays the distribution of genotype calls for each sample (one stack = one sample). Homozygous reference (allele `A`) or alternate (allele `B`) calls are shown in blue and green, respectively; heterozygous (`H`) calls in orange; and no-calls (`N`) in red. The y-axis is in absolute number of markers, not a proportion.

The bottom panel summarizes the array-wide distribution of intensity values for each sample. Each colored line connects a specific quantile (say, the 25th percentile) across samples. Samples are sorted left-to-right by median intensity, and the sorting order is the same in the top and bottom panels. Samples flagged as low-quality are indicated with an open dot along the baseline of the top panel.

The single failed array in this batch stands out both for its excess of no-calls and for its very wide intensity distribution.

Interpreting intensity distributions

Let's inspect the intensity distribution of the failed array and a couple of good arrays more closely, using the `intensityhist()` function:

```
bad <- which( is.filtered(geno)$samples ) # see ?is.filtered for details
good <- which( !is.filtered(geno)$samples )
intensityhist(geno[ ,c(bad, good[1:2]) ])
```

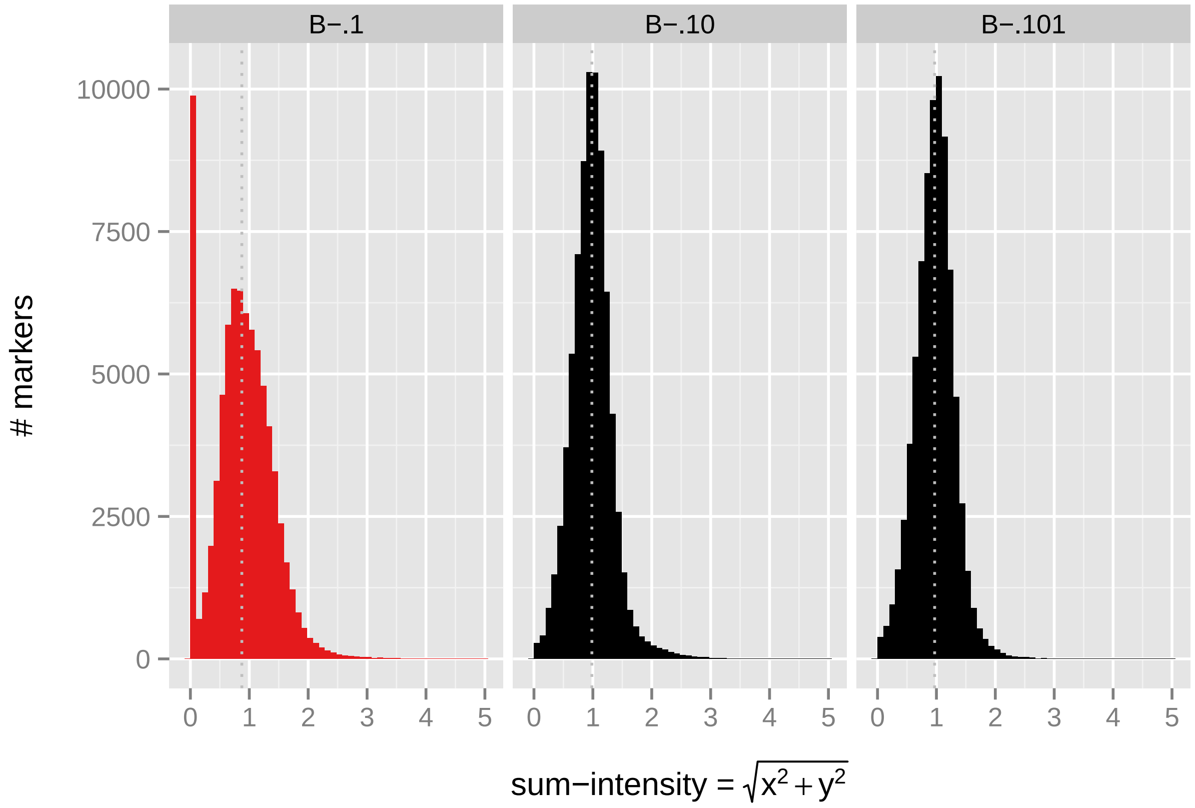

The rightmost panel is the intensity distribution from the failed array. (Because this sample has filters set, `intensityhist()` draws it in red.) Relative to the two good arrays, its distribution is slightly skewed towards lower values, and it has a very large spike near zero. Markers for which there is very low hybridization signal will yield no-calls. Note that the distributions for the good arrays are bell-shaped and approximately symmetric, with mean around one. This is a property of Illumina's affine-polish algorithm (Steemers *et al.* 2006).

The consistency of the shape of the intensity distribution for good arrays suggests that we can perform a statistical test for the "goodness" of hybridization for a sample. `argyle` implements a Kolmogorov-Smirnov test (KS test) which compares the shape of the intensity distribution for each sample in a `genotypes` object to a reference vector computed either from known good samples or by randomly sampling from a target distribution (Didion *et al.* 2014). For sake of simplicity we will use random draws from $N(1, 0.5)$ as the target distribution.

```
ref <- rnorm(1000, mean = 1, sd = 0.5)
ks.result <- intensity.vs.ref(geno, ref)

# what is the value for the bad sample?
ks.result[bad]
```

```
##        B-.1
## 0.02762719
```

```
# plot histogram of K across all samples
hist(ks.result, col = "grey", border = NA,
     xlab = "KS test statistic", ylab = "frequency",
     main = NULL)
```

```r
# show position of the bad sample
abline(v = ks.result[bad], lty = "dashed", col = "black")
```

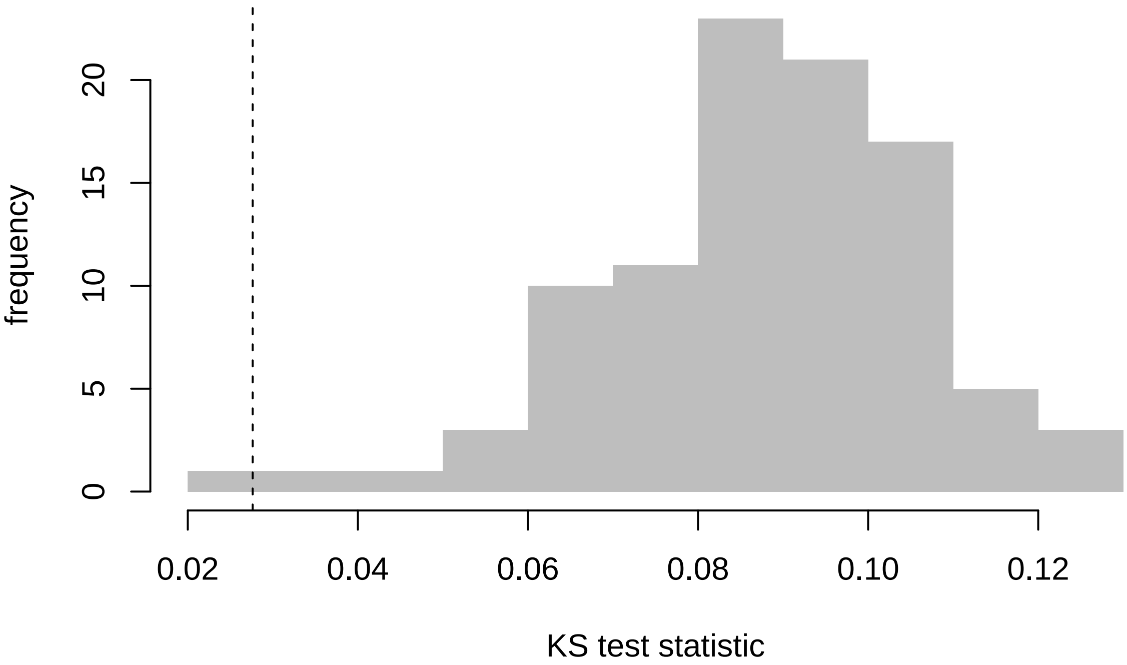

The `intesity.vs.ref()` function returns K, the test statistic from a two-sided KS test. See that the bad sample, which has an excess of markers for which there is low signal, falls in the lower tail of the distribution of K for the 96 samples in this dataset.

As we have concinced ourselves that sample B-.1 represents a failed array, we can drop it from the dataset in order to exclude it from future analyses. To drop samples flagged as low-quality, do

```r
geno.clean <- apply.filters(geno, "samples")
```

```
## Dropping 0 markers and 1 samples...
```

```r
summary(geno.clean)
```

```
## --- geno.clean ---
## A genotypes object with 77808 sites x 95 samples
## Allele encoding: 01
## Intensity data: yes (raw)
## Sample metadata: yes ( 0 male / 0 female / 95 unknown )
## Filters set: 0 sites / 0 samples
```

The resulting `genotypes` object now has only 95 samples, and all filters have been reset.

Marker-level QC

With a set of high-quality genotypes in hand we can proceed to quality checks on individual markers. Filtering criteria are of course critically dependent on the array platform, the population from which samples are

drawn and the experimental questions. Most analyses will benefit from removing markers with an excess of no-calls, as these bring little information. Markers with an excess of heterozygous calls are also likely to be low-performing.

To get a sense of the distribution of calls per marker, we can do

```
calls <- summarize.calls(geno.clean, "marker")
head(calls)
```

```
##                  marker  A  B  H N
## UNC6               UNC6 30 21 43 1
## JAX00000010 JAX00000010 30 22 43 0
## JAX00240603 JAX00240603 18 37 40 0
## JAX00240610 JAX00240610 95  0  0 0
## JAX00240613 JAX00240613 30 22 43 0
## JAX00240636 JAX00240636 67  5 23 0
```

This returns a table with counts of `A`, `B`, `H` and `N` calls per marker. We can inspect the distribution of missingness to choose an appropriate threshold.

```
hist(calls$N, col = "grey", border = NA,
     xlab = "count of no-calls", ylab = "frequency",
     main = NULL)
```

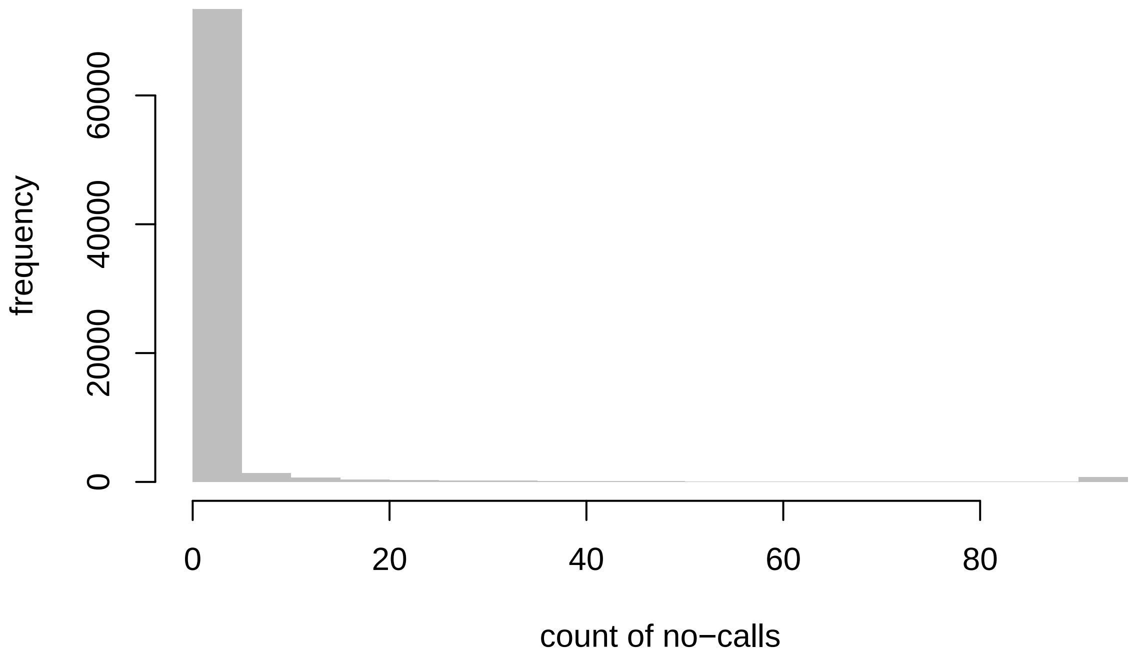


```
hist(with(calls, A+B), col = "lavender", border = NA,
     xlab = "count of homozygous calls", ylab = "frequency",
     main = NULL)
```

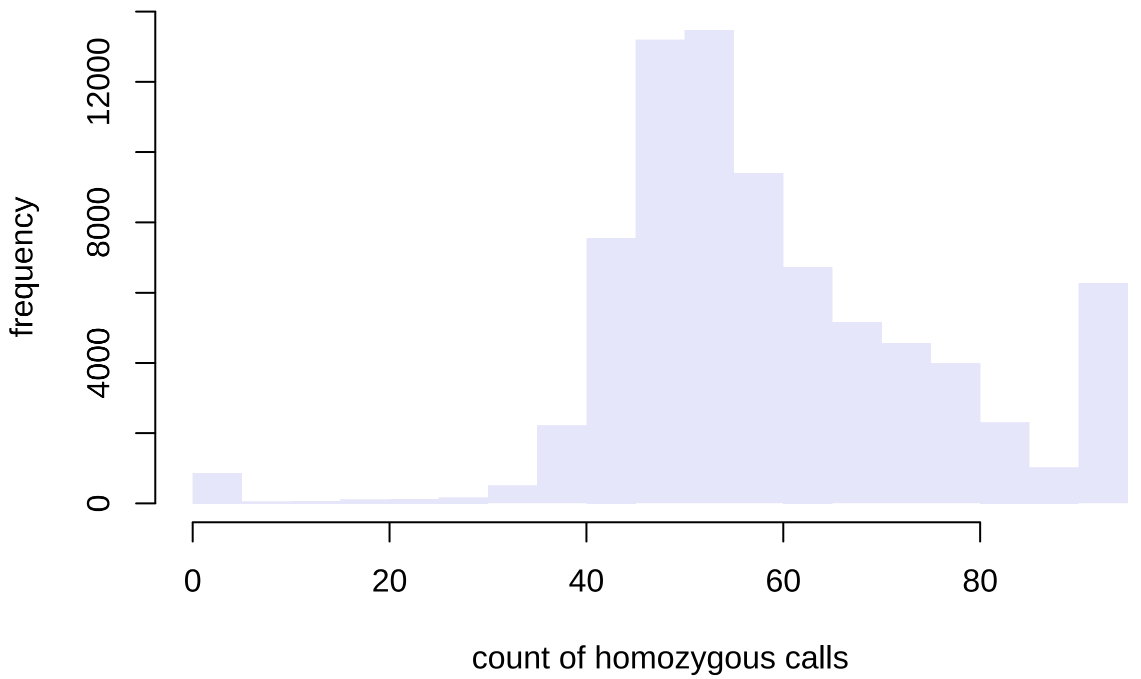

Clearly most markers have a relatively small number of no-calls. Thresholds of at most 20 no-calls and at least 30 homozygous calls are reasonable.

Marker-level checks are wrapped in the function `run.marker.qc()`. Important arguments are

- `max.N` – upper threshold for count of no-calls per marker
- `max.H` – upper threshold for count of heterozygous calls per marker
- `min.hom` – lower limit (inclusive) for count of homozygous calls per marker

```
geno.clean <- run.marker.qc(geno.clean, max.N = 20, min.hom = 30)
```

```
## Performing QC checks on genotype calls per marker...
## 2327 markers and 0 samples now flagged as low-quality.
```

Of 77808 markers on the array, 2327 (or 3%) fail our cursory filters. Apply the filters to get a final, clean dataset.

```
geno.final <- apply.filters(geno.clean)
```

```
## Dropping 2327 markers and 0 samples...
```

Biologically-motivated QC

The quality checks described so far are mostly technical in nature. Many experiments will offer biological constraints that can be exploited as additional quality checks.

Relatedness

In the present case, the 75481 outbred mice which pass QC are expected to to be approximately equally related. We can check that this is true by constructing a genetic relationship matrix (or "kinship matrix") for the samples and checking for gross population structure. `argyle` provides fast calculation of the proportion of alleles shared identical-by-state (IBS) between samples using `dist()`, and an accompanying visualization via `heatmap()`.

```
K <- dist(geno.final[,31:50])
```

```
## Computing distance matrix...
```

```
dim(as.matrix(K)) # should be 20x20
```

```
## [1] 20 20
```

```
heatmap(geno.final[,31:50], d = K)
```

```
## Nothing to do; genotypes already in requested coding.
## Using user-supplied distance matrix.
## Rendering heatmap...
```

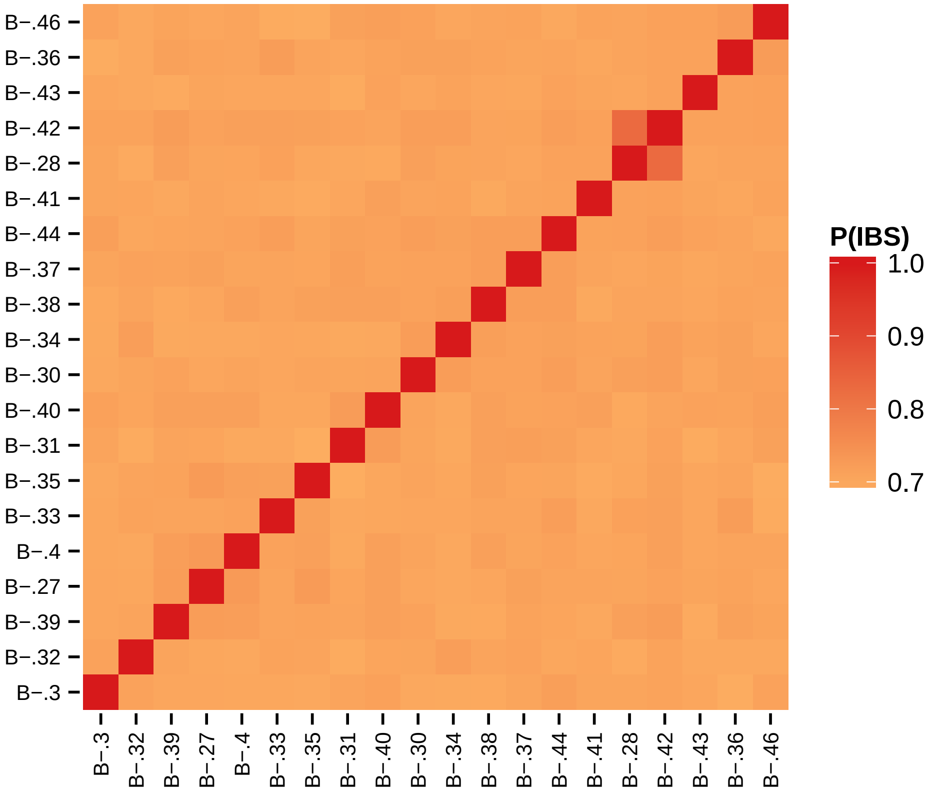

The heatmap representation of the kinship matrix is hierarchically clustered so that more related samples are adjacent to each other. As expected, self-similarity (entries on the diagonal) is 1, and most off-diagonal entries – except perhaps for samples B.-42 and B.-28 – have similar values. We can conclude that the population has, at most, very weak structure.

Sex-chromosome concordance

A second biological sanity check is for concordance between the true sex of each sample (if known) and calls on the X and Y chromosomes. (The following assumes that samples are from a diploid species with XY sex chromosome system, with males the heterogametic sex.) First, check the nominal sex of the samples in our example dataset using the `sextable()` shortcut function:

```
sextable(geno.final)
```

```
##    male  female unknown
##       0       0      95
```

It turns out that the sex of these animals is unknown, but we can predict it based on the number of homozygous (`A` or `B` allele) calls on the Y chromosome. For the MegaMUGA array, male samples will have at least 30 good calls on chrY and females will have at most 10.

```
my.sexes <- predict.sex(geno.final, min.AB = 30)
```

```
## Predicting sex using count of good calls on chrY...
```

```
table(my.sexes$predicted)
```

```
##
##  0  1
##  1 94
```

Since 0=unknown sex and 1=male, we can see that 94 samples appear to be male and the remaining 1 are still unknown. No samples appear to be female.

References

Didion, J. P., R. J. Buus, Z. Naghashfar, D. W. Threadgill, and H. C. Morse*et al.*, 2014 SNP array profiling of mouse cell lines identifies their strains of origin and reveals cross-contamination and widespread aneuploidy. BMC Genomics 15: 847.

Steemers, F. J., W. Chang, G. Lee, D. L. Barker, and R. Shen*et al.*, 2006 Whole-genome genotyping with the single-base extension assay. Nat Meth 3: 31–33.
