# Supplementary material for: argyle: An R Package for Analysis of Illumina Genotyping Arrays
Source: G3 (Bethesda). 2015 Dec 18;6(2):281–6. doi: 10.1534/g3.115.023739 (PMC4751548; doi:10.1534/g3.115.023739)
Supplement: Supporting Information [file supp_g3.115.023739_FileS4.pdf]

Analysis of an experimental cross `argyle`

Andrew P Morgan

2015-10-10

Introduction

An important use of genotyping arrays is for genotyping offspring of an experimental cross (say, an F_2 or backcross) as a precursor to QTL mapping. This vignette demonstrates a complete analysis, from raw array genotypes to candidate QTL. Data are taken from a backcross of a recombinant inbred mouse strain from the Collaborative Cross population, CC011/Unc, to the C57BL/6J strain for mapping of a spontaneuous colitis phenotype (Rogala *et al.* 2014). The `R/qtl` (Broman *et al.* 2003) package is used for QTL mapping.

Analysis of an experimental cross usually proceeds in five steps.

1. Perform quality checks on genotype calls
2. Identify informative markers, using the "clean" dataset
3. Confirm pedigree relationships, using the informative markers
4. Convert to `R/qtl` format
5. Perform QTL mapping with `R/qtl`

The dataset used in this vignette has already been converted to a `genotypes` object. Load it and `argyle` into the `R` session.

```
library(argyle)
load("datasets/colitis.Rdata")
```

Step 1: quality checks

First inspect the contents of the `genotypes` object:

```
summary(geno)
```

```
## --- geno ---
## A genotypes object with 77808 sites x 116 samples
## Allele encoding: native
## Intensity data: yes (raw)
## Sample metadata: yes ( 63 male / 53 female / 0 unknown )
## Filters set: 0 sites / 0 samples
```

The dataset contains 77808 samples in total, of which 3 are CC011/Unc inbred individuals from the parental generation and 113 are from the N_2 generation of the cross. First, we will use the sample names to assign them to the parental or N_2 generations.

Convert genotypes to numeric encoding to speed up quality checks.

```
geno <- recode(geno, "01")
```

```
## Recoding to 0/1/2 using reference alleles.
```

Now run the standard sample-level QC and produce the QC summary plot. Allow up to 4000 no-calls and 20000 heterozygous calls.

```
geno <- run.sample.qc(geno, max.N = 4e3, max.H = 20e3)
```

```
## Performing QC checks on genotype calls...
## Performing QC checks on hybridization intensities...
## 0 markers and 1 samples now flagged as low-quality.
```

```
qcplot(geno)
```

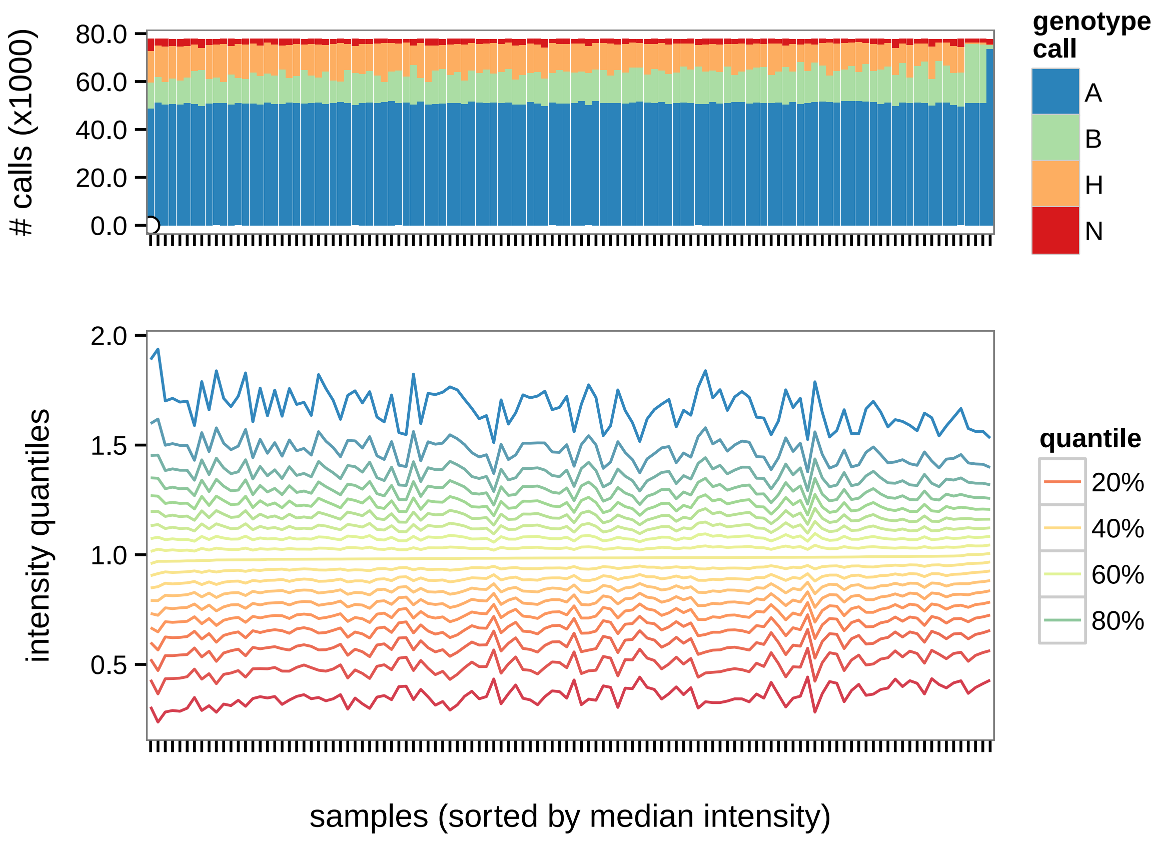


```
summarize.filters(geno)
```

```
##   sites samples
## N     0       1
## H     0       0
## I     0       0
## F     0       0
```

A single sample is of borderline quality due to an excess of no-calls. We will retain it for now.

Step 2: identifying informative markers

A marker is informative in this cross if:

- the two parental strains have opposite homozygous genotypes
- genotype frequencies in tehe N_2 progeny are in the expected range (~ 0.5)

A single CC011/Unc male (`UNC_arM001`) sired all the F_1 and N_2 progeny in this cross. To find informative markers, choose those which are homozygous for opposite alleles in that animal and the (representative) C57BL/6J animal in the dataset. The function `fixed.diffs()` does just that.

```
b6 <- grep("^C57BL\\/6J", colnames(geno), value = TRUE)
dad <- "UNC_arM001"
infm <- fixed.diffs(geno[ ,c(b6,dad) ])

# how many informative markers?
sum(infm)
```

```
## [1] 22492
```

Next identify markers which have outlying proportion of heterozygous calls across the N_2 offspring, as these probably represent genotyping errors.

```
hets <- heterozygosity(geno)
hist(hets, col = "grey", border = NA, main = NULL,
     xlab = "proportion of N2s heterozygous", ylab = "frequency")
```

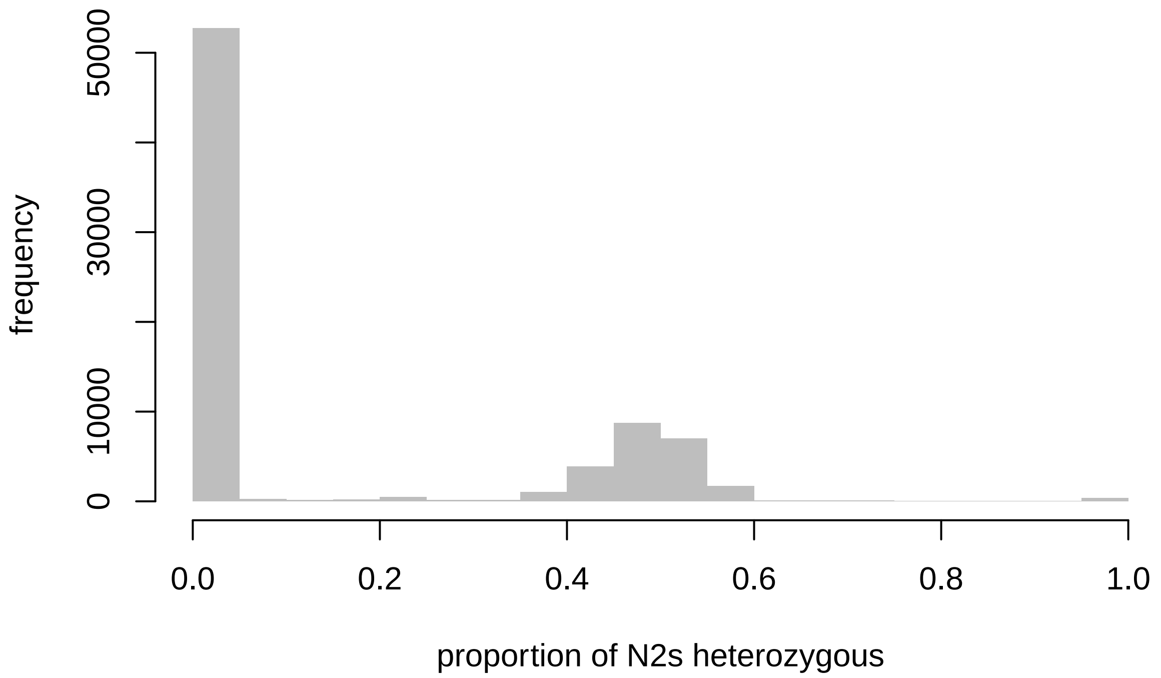

Clearly many markers are not informative in this cross (the spike in the distribution at zero), which we already knew. The mode in the distribution around 0.5 represents the markers we want to keep.

Now keep just the informative markers with an "appropriate" level of heterozygosity. (Note that we could have applied a formal test to the genotype frequencies, instead of this heuristic, but the difference is likely to be negligible.)

```r
geno.final <- geno[ infm & (hets > 0.3 & hets < 0.7), ]
print(geno.final)
```

```
## --- gty ---
## A genotypes object with 21726 sites x 116 samples
## Allele encoding: 01
## Intensity data: yes (raw)
## Sample metadata: yes ( 63 male / 53 female / 0 unknown )
## Filters set: 0 sites / 1 samples
##
## Counts of markers by chromosome:
##  chr1  chr2  chr3  chr4  chr5  chr6  chr7  chr8  chr9 chr10 chr11 chr12
##  2205  1686  1398  1411   794  1218  1394  1640   288   951  1530  1637
## chr13 chr14 chr15 chr16 chr17 chr18 chr19  chrX  chrP
##   368  1122  1107  1039  1217   713     2     4     2
```

See that there remain markers on all chromosomes at more than sufficient density for a smallish backcross.

Step 3: confirmation of sample identity

All the N_2 offspring from a backcross are, in expectation, equally related. We can check the kinship matrix to identify potentially pairs of individuals which are much more closely-related than expected; these may represent duplicated samples, or breeding errors.

```r
n2 <- subset(geno.final, fid == "N2", by = "samples")
heatmap(n2)
```

```
## Nothing to do; genotypes already in requested coding.
## Computing distance matrix...
## Rendering heatmap...
```

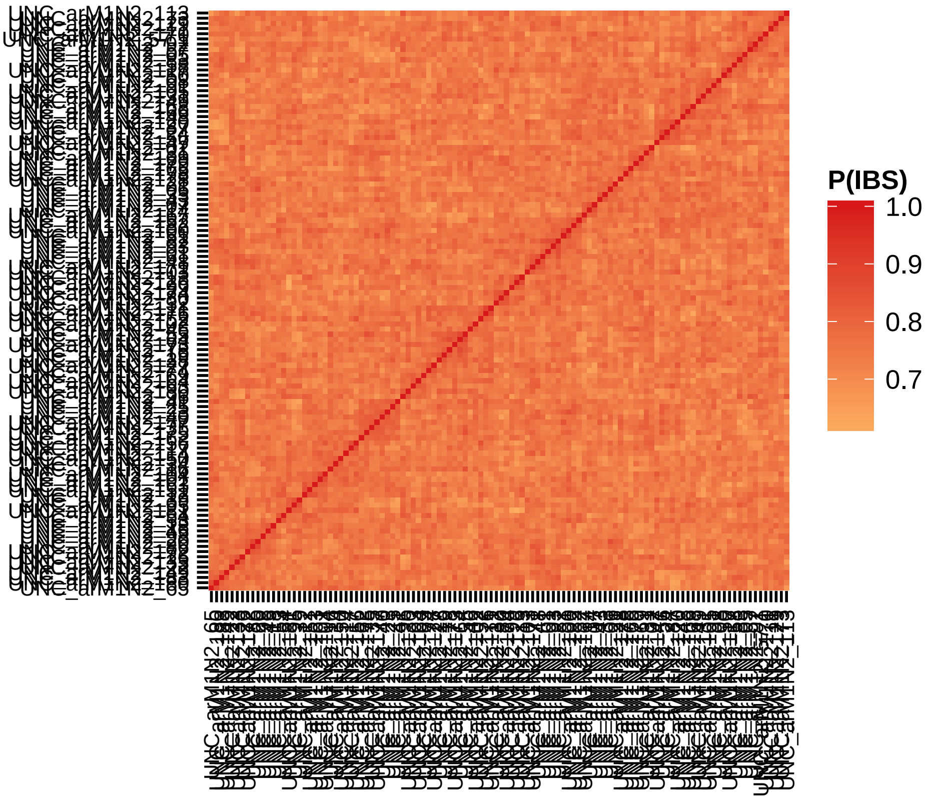

A second sanity check is the proportion of heterozygous calls per sample. (Contrast this to the per-marker heterozygosity calculated previously.) This should have an approximately normal distribution in the N_2 progeny, with mean ~ 0.5.

```
ihet <- prop.het(n2)
hist(ihet, col = "grey", border = NA, main = NULL,
     xlab = "proportion of markers heterozygous", ylab = "frequency")
```

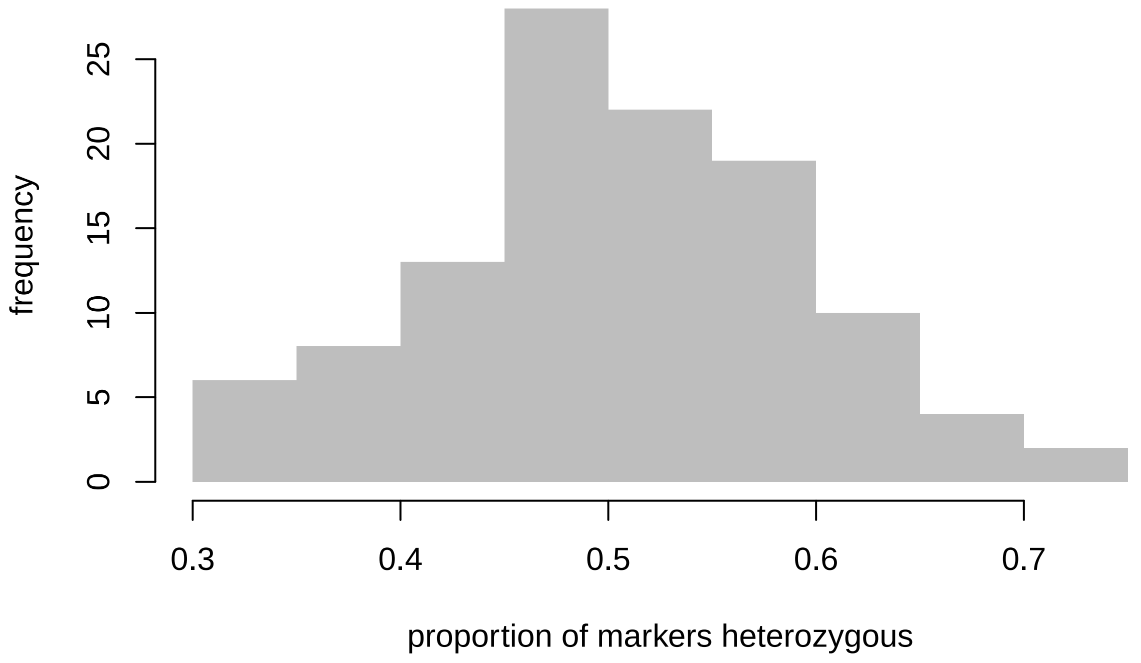

Step 4: conversion for R/qtl

Before exporting to R/qtl format, an addition recoding of the genotypes is required. R/qtl, and most other software designed for the analysis of traditional cross designs, expects genotypes to be coded with respect to teh parental lines of the cross, not with respect to an arbitrary reference. We can achieve this in argyle using the recode.to.parent() function. This function recodes genotypes as the count of alleles shared identical-by-state with a chosen reference individual (in our case, the sire/grandsire of the cross, UNC_arM001.)

```
geno.recoded <- recode.to.parent(geno.final, "UNC_arM001")
```

Some complications will arise if the reference individual is heterozygous, but we won't encounter this because (1) the parents of our cross are inbred lines; and (2) we have pruned away markers with any evidence of residual heterozygosity.

Finally, load R/qtl and perform the conversion.

```
library(qtl)
```

```
##
## Attaching package: 'qtl'
##
## The following object is masked from 'package:argyle':
##
##     replace.map
```

```
cross <- as.rqtl( subset(geno.recoded, fid == "N2", by = "samples"),
                  type = "bc" )
```

```
## Exporting genotypes at 21724 markers on 20 chromosomes.
## Converting genotypes...
## Done.
```

```
summary(cross)
```

```
## Warning in summary.cross(cross): Some markers at the same position on chr
## 1,2,3,4,5,6,7,8,9,10,11,12,13,14,15,16,17,18,19,X; use jittermap().

## Warning in summary.cross(cross): Invalid genotypes on X chromosome:
##     Observed genotypes: 1 2 3

##     F2 intercross
##
##     No. individuals:    112
##
##     No. phenotypes:     6
##     Percent phenotyped: 99.1 100 100 100 100 100
##
##     No. chromosomes:    20
##         Autosomes:      1 2 3 4 5 6 7 8 9 10 11 12 13 14 15 16 17 18 19
##         X chr:          X
```

```
##
##     Total markers:      21724
##     No. markers:        2205 1686 1398 1411 794 1218 1394 1640 288 951 1530
##                         1637 368 1122 1107 1039 1217 713 2 4
##     Percent genotyped:  99.1
##     Genotypes (%):
##           Autosomes:        AA:0.0       AB:50.5      BB:49.5  not BB:0.0
##                         not AA:0.0
##        X chromosome:        AA:0.0       AB:23.3      AY:66.2      BY:10.5
```

Several warnings are generated by `R/qtl`. Some are important; some are not.

- `Some markers at same position ...` – often safe to ignore. Marker positions in `R/qtl` are specified in centimorgans. Genetic (centimorgan) positions for markers on SNP arrays are typically obtained by linear interpolation from a sparser genetic map, and may be rounded to reflect the limited precision of this interpertation. `R/qtl` emits a warning but adds random noise to ensure that every marker has a unique position (and, therefore, that each inter-marker interval has a nonzero probability of containing a recombinant in the cross.)
- `Invalid genotypes on X chromosome` – worth further inspection. Impossible genotypes on the X chromosome represent either poorly-performing markers or misspecified sample sexes.

Step 5: QTL mapping

So far we have ignored the phenotypes included with this dataset. The phenotype is a histopathlogical score, taking values between 0 (no disease) and 21 (profound disease) inclusive. Before proceeding to QTL mapping, we can inspect the distribution of the phenotype in the N_2s.

```
hist( subset(samples(geno.recoded), fid == "N2")$pheno, main = NULL,
      col = "grey", border = NA, xlab = "score", ylab = "frequency" )
```

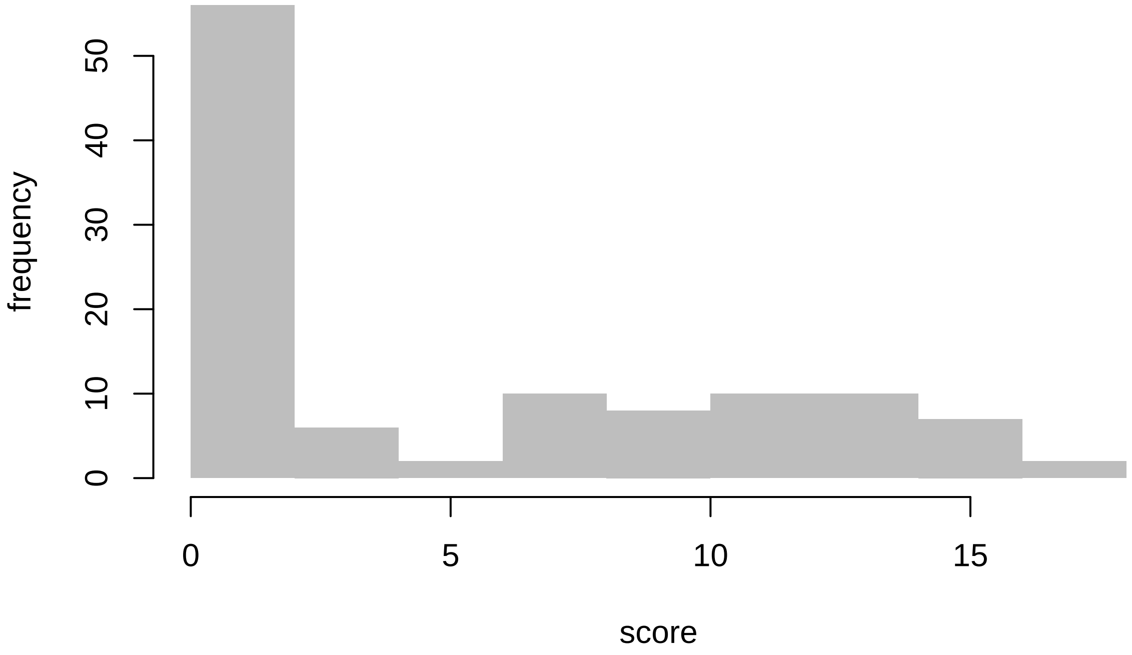

Many individuals are unaffected, and of those affected, the severity of disease is variable. As a first approximation we can map the histopathological score as if it is a continuous trait.

```
qtls <- scanone(cross)
```

```
## Warning in checkcovar(cross, pheno.col, addcovar, intcovar, perm.strata, : Dropping 1 individuals wi
```

```
## Warning in scanone(cross): First running calc.genoprob.
```

```
plot(qtls)
```

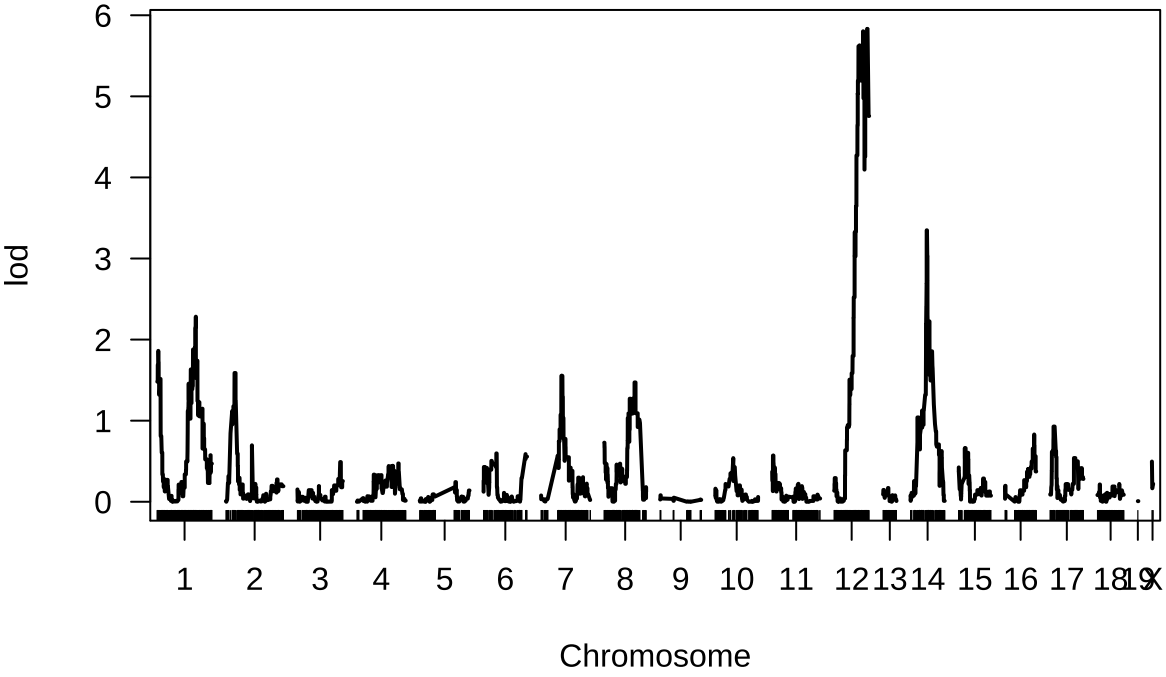

As reported in (Rogala *et al.* 2014), there are QTL on chromosomes 1, 12 and 14.

For further discussion of QTL mapping with `R/qtl`, consult one of the excellent tutorials which accompany that package.

References

Broman, K. W., H. Wu, S. Sen, and G. A. Churchill*et al.*, 2003 R/qtl: QTL mapping in experimental crosses. Bioinformatics 19: 889–890.

Rogala, A. R., A. P. Morgan, A. M. Christensen, T. J. Gooch, and T. A. Bell*et al.*, 2014 The collaborative cross as a resource for modeling human disease: CC011/Unc, a new mouse model for spontaneous colitis. Mamm Genome 25: 95–108.
