# Supplementary material for: argyle: An R Package for Analysis of Illumina Genotyping Arrays
Source: G3 (Bethesda). 2015 Dec 18;6(2):281–6. doi: 10.1534/g3.115.023739 (PMC4751548; doi:10.1534/g3.115.023739)
Supplement: Supporting Information [file supp_g3.115.023739_FileS5.pdf]

Population-based analyses with argyle

Andrew P Morgan

2015-10-10

Introduction

In this vignette we introduce `argyle` utilities for analyzing genotypes from natural populations. We make use of a dataset containing genotypes for 28 wild-caught mice, representing the three subspecies of *Mus musculus*, from the Mouse Diversity Array (Yang *et al.* 2011). The MDA is a high-density Affymetrix array; for sake of demonstration we use only the 14306 markers on chromosome 19.

This vignette also provides an overview of utilities for interacting with genotypes stored in the `PLINK` (Purcell *et al.* 2007) binary format. `PLINK` is widely used software with many utilities for management of genetic datasets and association studies. Although `PLINK` accepts many input formats, they are all converted to a space-efficient binary intermediate prior to analysis. The `PLINK` binary fileset consists of three parts distinguished by their suffix:

- `*.bed` – biallelic genotype data, stored in two bits per genotype
- `*.bim` – marker map
- `*.fam` – "family file" with sample identifiers, pedigree information (if applicable) and other sample metadata

For a more detailed description see the [PLINK documentation].

```
library(argyle)
```

The PLINK interface

`argyle` can use genotypes stored in `PLINK` format in two ways: either by reading them into the `R` session as a `genotypes` object, or by maintaining a pointer to the `PLINK` fileset on disk without holding genotypes themselves in memory. The latter is useful when working with large datasets that do not fit comfortably in RAM.

To load data from a `PLINK` fileset as a `genotypes` object, use `read.plink()`. This function takes a single argument, `prefix`, and expects to find three files: `prefix.bed`, `prefix.bim` and `prefix.fam`.

```
wilds <- read.plink("datasets/wild.chr19")
```

```
## Reading family info from: <datasets/wild.chr19.fam>
## Reading marker info from: <datasets/wild.chr19.bim>
## Reading binary genotypes from: <datasets/wild.chr19.bed>
```

```
summary(wilds)
```

```
## --- wilds ---
## A genotypes object with 14306 sites x 28 samples
## Allele encoding: 01
## Intensity data: no
## Sample metadata: yes ( 13 male / 15 female / 0 unknown )
## Filters set: 0 sites / 0 samples
```

```
table(samples(wilds)$fid)
```

```
##
## cas dom mus
##  10   9   9
```

Genotypes are read in the `01` encoding: numeric, as counts of the non-reference allele.

If we prefer to keep the data out of memory, we can instead create a pointer to the fileset using `plinkify()`:

```
ptr <- plinkify("datasets/wild.chr19")
print(ptr)
```

```
## -- Pointer to a PLINK fileset --
## Source: /Users/apm/Dropbox/pmdvlab/argyle/manuscript/vignettes/datasets/wild.chr19.bed
## Ouput dir: /private/var/folders/_r/xn9svcws2sv9xns429lr0x_00000gp/T/RtmpzDa55Y
```

This pointer keeps track of both the location of the target fileset, and the location of a scratch space where any intermediate files created by calls to `PLINK` utilities – and `PLINK` generates *lots* of such files – are stored, to avoid cluttering the working directory. By default `argyle` uses the `R` session's temporary file directory as scratch space. Contents of that directory are wiped when the `R` session is terminated.

The `argyle` package also provides wrapper functions for many `PLINK` utilities. Functions in this family all have the suffix `*plink`. They are executed as command-line calls and assume that an executable named `plink` (or a link by the same name) is in the user's path. With the exception of those marked with an asterisk below, they will work with either `PLINK` v1.0.x or the more recent (and *much* faster) `PLINK` v1.9+.

- `mds.plink` – classical multidimensional scaling (MDS) for visualizing population structure
- `pca.plink` – principal components analysis (PCA) for visualizing population structure
- `ld.plink` – pairwise linkage disequilibrium (LD) calculations
- `prune.plink` – pruning of markers based on local LD
- `filter.plink` – general-purpose filtering based on allele frequency, Hardy-Weinberg equilibrium, chromosome, position, blacklists, . . .
- `weir.fst.plink`* – calculation of between-population fixation indices (F_{st}) using Weir & Cockerham's estimator)
- `assoc.plink` – genotype-phenotype association tests under a variety of models
- `tdt.plink` – transmission disequilibrium test (TDT), a family-based association test

Population structure

Preliminary analyses of patterns of relatedness between samples – that is, population structure – often make use of a dimension-reduction procedure such as principal components analysis (PCA) or multidimensional scaling (MDS). PCA, as applied to genotypes at biallelic markers, amounts to eigendecomposition of the (co)variance of allele counts at each marker. `argyle` provides a `pca()` method for `genotypes` objects, and an accompanying `plot()` function which produces an informative plot of the projection of samples onto PCs.

```
pc <- pca(wilds)
```

```
## Preparing input matrices...
##  replacing missing values with minor-allele frequency...
```

```
## Computing principal components of genotypes matrix...
##  (using base::prcomp() ...)
## Done.
```

```
plot(pc, screeplot = TRUE)
```

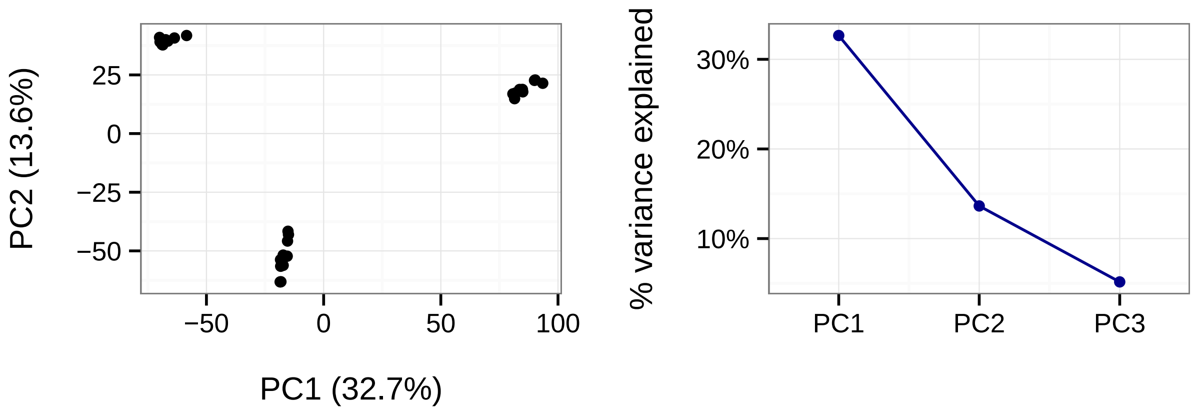

Missing values are substituted with the allele population allele frequency at the corresponding marker; when many genotypes are missing this will tend to cause the points to "contract" into a blob in the center of the subspace defined by the PCs.

Like all the plotting functions in `argyle`, the `plot()` method for PCA results uses `ggplot2`. The plot it returns can be customized and extended like any `ggplot` object. For example, we can produce a more polished figure by suppressing the screeplot (by omitting `screeplot = TRUE`) and coloring the points by the value of `fid` (sample group), which in this case corresponds to their subspecies of origin.

```
library(ggplot2)
cols <- c("dom" = "blue", "mus" = "red", "cas" = "green")
groups <- c("dom" = "M. m. domesticus", "mus" = "M. m. musculus",
            "cas" = "M. m. castaneus")
# show = "nothing" draws just the skeleton of the plot,
#   so we can add our own layers for finer control
plot(pc, show = "nothing") +
    geom_point(aes(colour = fid)) +
    scale_colour_manual("subspecies", values = cols,
                        breaks = names(groups),
                        labels = groups) +
    theme(legend.text = element_text(face = "italic"))
```

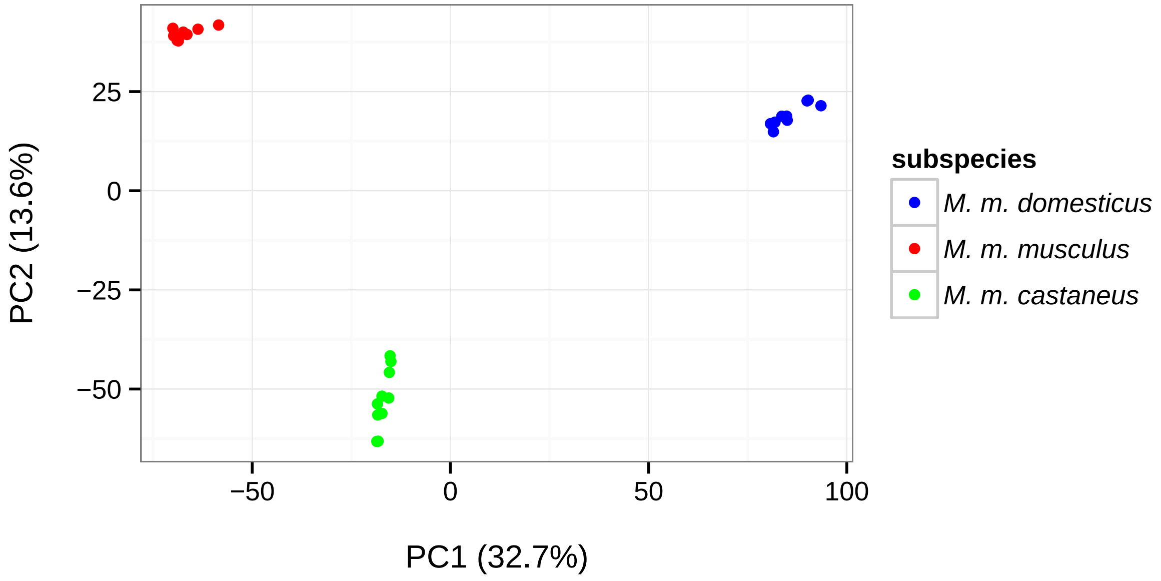

Compare this to the result obtained by multidimensional scaling (MDS) using `PLINK`.

```
mds <- mds.plink(ptr)
ggplot(mds) +
    geom_point(aes(x = MDS1, y = MDS2, colour = fid)) +
    scale_colour_manual("subspecies", values = cols,
                        breaks = names(groups),
                        labels = groups) +
    theme_bw() + theme(legend.text = element_text(face = "italic")) +
    coord_equal()
```

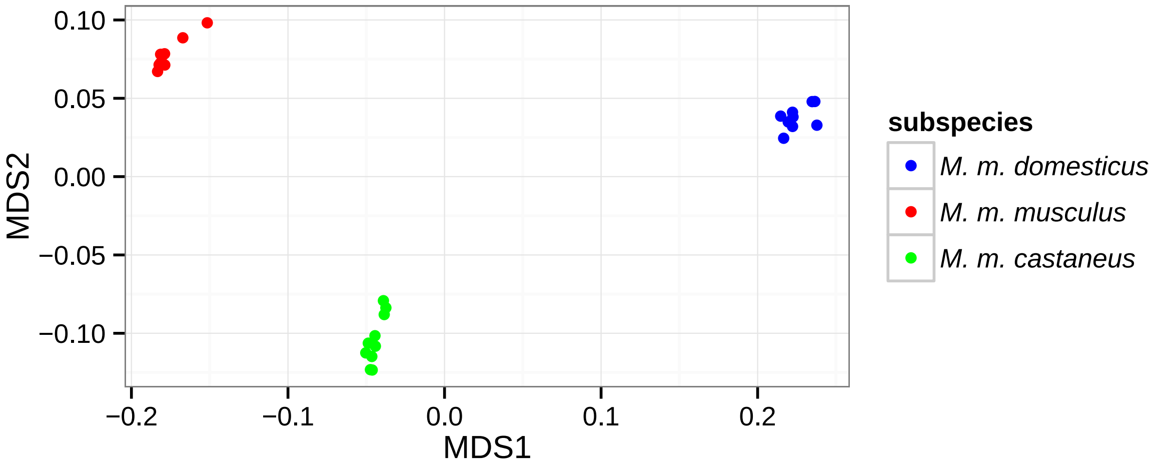

MDS and PCA give, as expected, qualitatively similar results that support a clear genetic differentiation of the present samples according to subspecies of origin.

Allele frequencies

Obtain non-reference allele counts with `freq()` and plot the allele-frequency spectrum.

```r
f <- freq(wilds, "samples")
hist(f, breaks = seq(0,1,0.025),
     col = "grey", border = NA,
     main = NULL, xlab = "allele frequency bin",
     ylab = "frequency")
```

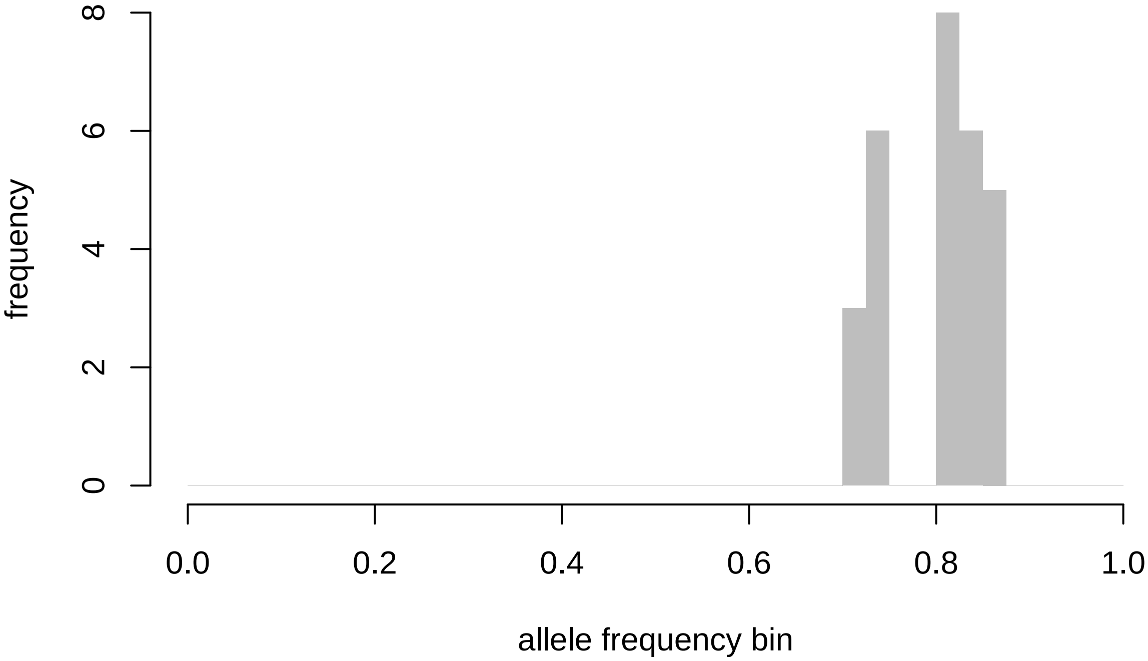

In working with genotypes from natural populations it may be advantageous to recode genotypes in terms of the count of the *minor allele* rather than the *non-reference allele.* This can be done with `recode()`:

```r
wilds.recode <- recode(wilds, "relative")
```

```
## Recoding to 0/1/2 using empirical frequencies.
```

```r
summary(wilds.recode)
```

```
## --- wilds.recode ---
## A genotypes object with 14306 sites x 28 samples
## Allele encoding: relative
## Intensity data: no
## Sample metadata: yes ( 13 male / 15 female / 0 unknown )
## Filters set: 0 sites / 0 samples
```

> **Caution:** once alleles are recoded as major/minor within a dataset, they cannot be unambiguously reverted to the reference/alternative encoding.

Now inspect the allele-frequency spectrum again. It should have closer to the expected L-shape. Note that this is now a *folded* AFS since values are constrained to fall on the interval $[0, \frac{1}{2}]$.

```r
f <- freq(wilds.recode, "samples")
hist(f, breaks = seq(0,0.5,0.025),
     col = "grey", border = NA,
     main = NULL, xlab = "allele frequency bin",
     ylab = "frequency")
```

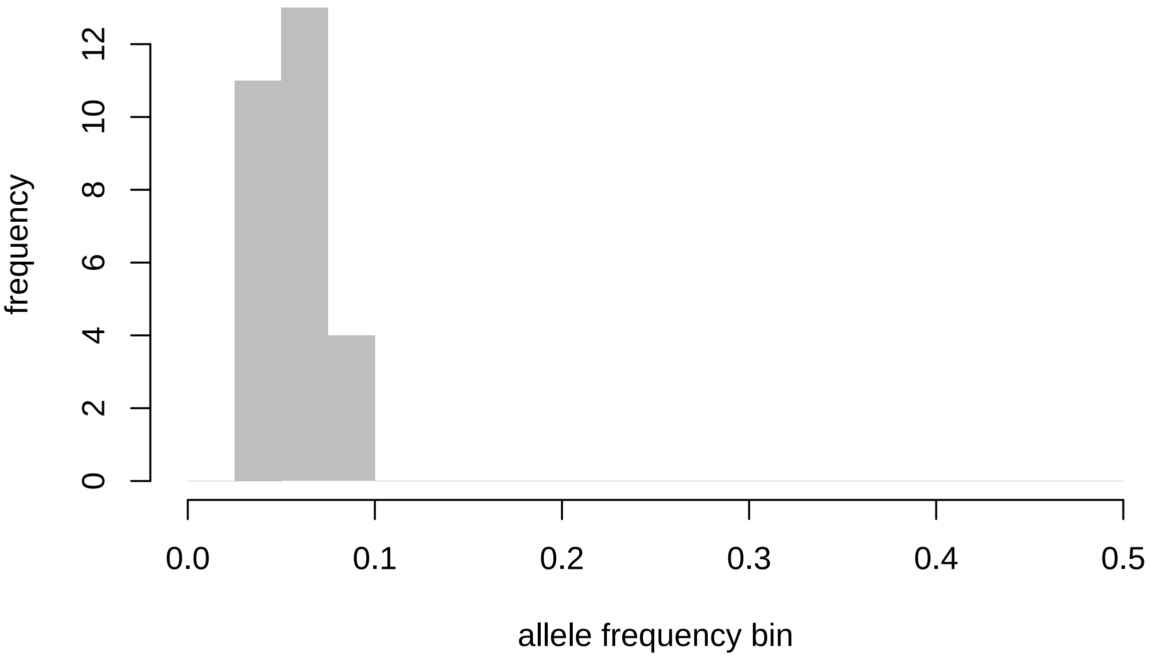

Population differentiation

F_{st} measures differentiation of alleles between populations – that is, the proportion of variance in allele frequencies which lies between versus within populations. The unbiased $\hat{F}_{st}$ estimator of Weir & Cockerham is implemented in `PLINK` and wrapped by `argyle`.

```r
fst <- weir.fst.plink(ptr)
```

```
## -- Pointer to a PLINK fileset --
## Source: /Users/apm/Dropbox/pmdvlab/argyle/manuscript/vignettes/datasets/wild.chr19.bed
## Ouput dir: /private/var/folders/_r/xn9svcws2sv9xns429lr0x_00000gp/T/RtmpzDa55Y
## [1] "--fst --family --keep-cluster-names cas dom "
## [1] "--fst --family --keep-cluster-names cas mus "
## [1] "--fst --family --keep-cluster-names dom mus "
```

```r
print(fst)
```

```
##          cas       dom       mus
## cas 0.550060 0.4957020 0.4916060
## dom 0.495702 0.6228444 0.6646920
## mus 0.491606 0.6646920 0.7161333
```

The return value is a (symmetric) matrix whose off-diagonal entries are F_{st} values and whose diagonal entries are the mean inbreeding coefficient in each population. Populations are assigned according to the value of `fid` in the sample metadata.

References

Purcell, S., B. Neale, K. Todd-Brown, L. Thomas, and M. A. Ferreira *et al.*, 2007 PLINK: A tool set for whole-genome association and population-based linkage analyses. Am J Hum Genet 81: 559–575.

Yang, H., J. R. Wang, J. P. Didion, R. J. Buus, and T. A. Bell *et al.*, 2011 Subspecific origin and haplotype diversity in the laboratory mouse. Nat Genet 43: 648–655.
